# NIR-II biomimetic nanoplatform optogenetic CD274 editing of HNSCC immunogenicity for enhanced photoimmunotherapy

**DOI:** 10.1016/j.mtbio.2026.102803

**Published:** 2026-01-13

**Authors:** Yang Chen, Longcai Liu, Xiaojuan Hu, Yilin Huang, Shijie Yao, Lichen Ji, Hai Zou, Xiaozhou Mou, Yu Cai

**Affiliations:** a Centre for Rehabilitation Medicine, Rehabilitation & Sports Medicine Research Institute of Zhejiang Province, Department of Rehabilitation Medicine, Cancer Centre, Zhejiang Provincial People's Hospital (Affiliated People's Hospital), Hangzhou Medical College, Hangzhou, Zhejiang, 310014, China; b Department of Emergency and Critical Care Medicine, Pudong New Area People's Hospital Affiliated to Shanghai Jiao Tong University School of Medicine, China; c Department of Joint Surgery, Shanghai East Hospital, Tongji University School of Medicine, Shanghai, 200092, China

**Keywords:** NIR-II, CRISPR/Cas9, Hsp70 promoter, Optogenetics, Head and neck squamous cell carcinoma

## Abstract

Although immunotherapy has achieved impressive breakthroughs in head and neck squamous cell carcinoma (HNSCC), it still encounters significant challenges such as the intrinsic low immunogenicity microenvironment and limited T cell infiltration. In this work, we aimed to edit the CD274 gene of HNSCC cells by optogenetics with second near-infrared (NIR-II) light, thereby reducing the CD274 expression and improving the efficacy of photo-immunogenic therapy. Specifically, a biomimetic nanoplatform (ARPC) was established by using an α-LDLR (low density lipoprotein receptor antibody) engineered red blood cell membrane (RBCm) to deliver NIR-II photothermal polymers and CRISPR/Cas9 plasmids. After intravenous injection into HNSCC-bearing mice, ARPC can induce heat stress upon NIR-II laser irradiation at tumor sites, causing the upregulation of Hsp70 to trigger CRISPR/Cas9 for CD274 editing. Moreover, the mild photothermal therapeutic effect of ARPC simultaneously induced immunogenic cell death in tumor cells for enhancing CD8^+^ T cell infiltration and proliferation, and thereby leading to photoimmunotherapy. This study provides an NIR-II optogenetic CRISPR/Cas9 CD274 for editing reprogrammed photo-immunogenic therapy strategy, showing great clinical potential for overcoming the low immunogenicity of HNSCC.

## Graphical abstract

**Figure:**
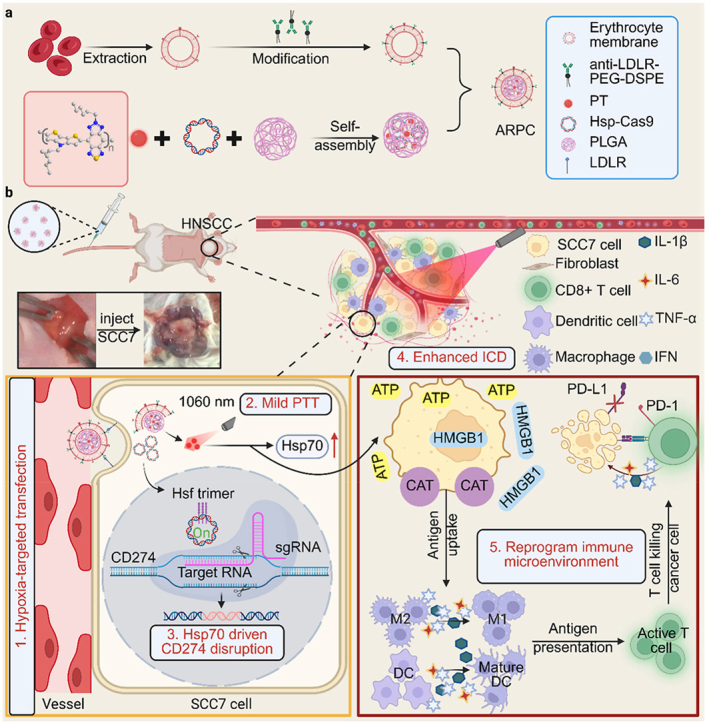

## 1 Introduction

Head and neck squamous cell carcinoma (HNSCC) is the sixth most common cancer globally, accounting for approximately 90 % of head and neck cancer cases [1,2]. The current standard treatment primarily relies on surgical resection followed by adjuvant chemotherapy and radiotherapy [3]. However, these conventional approaches are accompanied by significant side effects, including transient or permanent loss of speech, hearing impairment, difficulties in chewing and swallowing, and damage to healthy tissues such as the salivary glands, thyroid, and lymph nodes [4,5]. Immune checkpoint blockade (ICB) therapy has achieved impressive breakthroughs in HNSCC treatment and is considered the most promising approach for potentially eradicating the cancer [6,7]. Several monoclonal antibodies targeting CD274 have become the standard for treating various cancer types [[8], [9], [10]]. Nonetheless, since CD274 is widely distributed in normal tissues, their extensive blockade can result in severe immune related inflammatory responses, including immune mediated pneumonia and even myocarditis, and can also lead to hypothyroidism in some patients [[11], [12], [13]]. These severe side effects significantly impact the clinical efficiency of ICB therapy. HNSCC features inadequate T cell tumor infiltration and an immunosuppressive tumor microenvironment [[14], [15], [16]], often described as a “cold tumors”, which are key factors contributing to the poor response to ICB therapies [[17], [18], [19]]. Therefore, transforming a “cold” tumor microenvironment into a “hot” one has emerged as a promising strategy to enhance the benefits of current ICB therapies.

Clustered regularly interspaced short palindromic repeats associated protein 9 (CRISPR/Cas9), as a potent genome-editing tool, has recently been recognized for its substantial potential for disrupting CD274 ligand expression at the genetic level, presenting a novel approach for anti-CD274 cancer therapy [20,21]. Developing a safe and effective drug delivery method for CRISPR therapy had the potential to provide a wide range of treatments for genetic diseases. Currently, the most used in vivo delivery vectors are limited to polycationic liposomes and viral vectors [[22], [23], [24]]. Although viral delivery remains the most common method for in vivo CRISPR gene editing, viral vectors can trigger immune responses [25,26], carry the risk of genome integration, and may cause off-target DNA damage due to sustained expression of the gene editor [[27], [28], [29]]. If the potency and toxicity issues can be overcome, non-viral delivery strategies may address these limitations. To mitigate in vivo toxicity and immunogenicity, delivery vectors with high loading capacity and editing efficiency must be further optimized.

On the other hand, the timing of the initiation of CRISPR/Cas9 gene editing at a specific location is also a key issue. Mild photothermal therapy (MPTT) is a noninvasive oncological modality that selectively targets tumor tissue while minimizing damage to surrounding normal tissues and organs [[30], [31], [32]]. MPTT at mild temperatures often induces overexpression of heat shock proteins (Hsp) [[33], [34], [35], [36]], which could be a potential promoter. Furthermore, recent studies have demonstrated that MPTT can trigger immunogenic cell death (ICD) [37,38], releasing tumor-derived antigens for photo-immunogenic therapy [[39], [40], [41]]. Therefore, employing MPTT as a trigger can achieve site-specific and controllable gene editing while simultaneously stimulating photoimmunotherapy, leading to synergistic antitumor immunity.

**Scheme 1:**
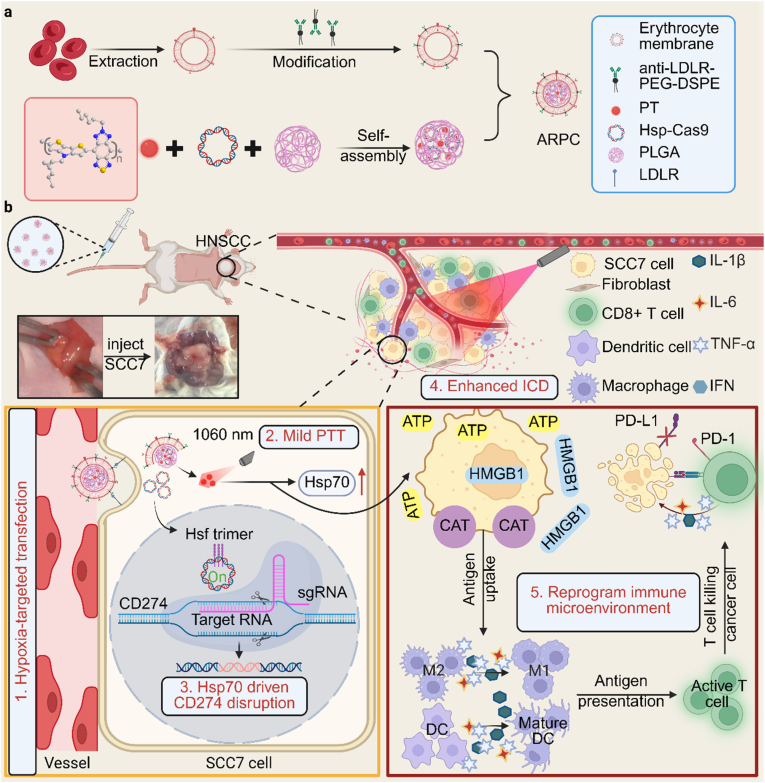
a) Illustrations of the α-LDLR-modified RBCm encapsulated Hsp70 promoter-driven CRISPR/Cas9 system (ARPC) preparation. b) Schematic representation of NIR-II optogenetic ARPC disruption in HNSCC cells and the antitumor immune responses that occurred after treatment.

In this work, we propose an Hsp70-driven CRISPR/Cas9 system for permanent disruption of the CD274 gene in HNSCC, which effectively reprograms the tumor immunosuppressive microenvironment to elicit multifaceted anti-cancer immune responses. As shown in Scheme 1, the Hsp70-driven CRISPR/Cas9 and organic conjugated polymers (PT) were encapsulated within α-LDLR-engineered red blood cell membranes (RBCm) [42] to construct the NIR-II optogenetic nanoplatform (ARPC). In this nanoplatform, the α-LDLR-modified RBCm serves as a biomimetic delivery vehicle, endowing ARPC with excellent biocompatibility, high drug encapsulation, high loading stability, and active targeting capability toward SCC7 cells. After intravenous injection into HNSCC mice, ARPC can effectively accumulate at tumor sites. Subsequently, upon NIR-II laser irradiation, PT induced thermal stress will lead to the upregulation of Hsp70 to promote CRISPR/Cas9, triggering the genome editing of CD274. Furthermore, MPTT induced ICD in tumor cells promoted dendritic cell (DC) maturation, and enhanced CD8^+^ T-cell infiltration. Overall, the proposed NIR-II optogenetic ARPC nanoplatform specifically targets tumor cells and permanently disrupts CD274 in tumor cells to reprogram the tumor microenvironment and induce ICD to prevent immune evasion, thereby achieving efficient photoimmunotherapy for HNSCC.

## 2 Results and discussion

### 2.1 Preparation and characterization of ARPC

**Fig. 1:**
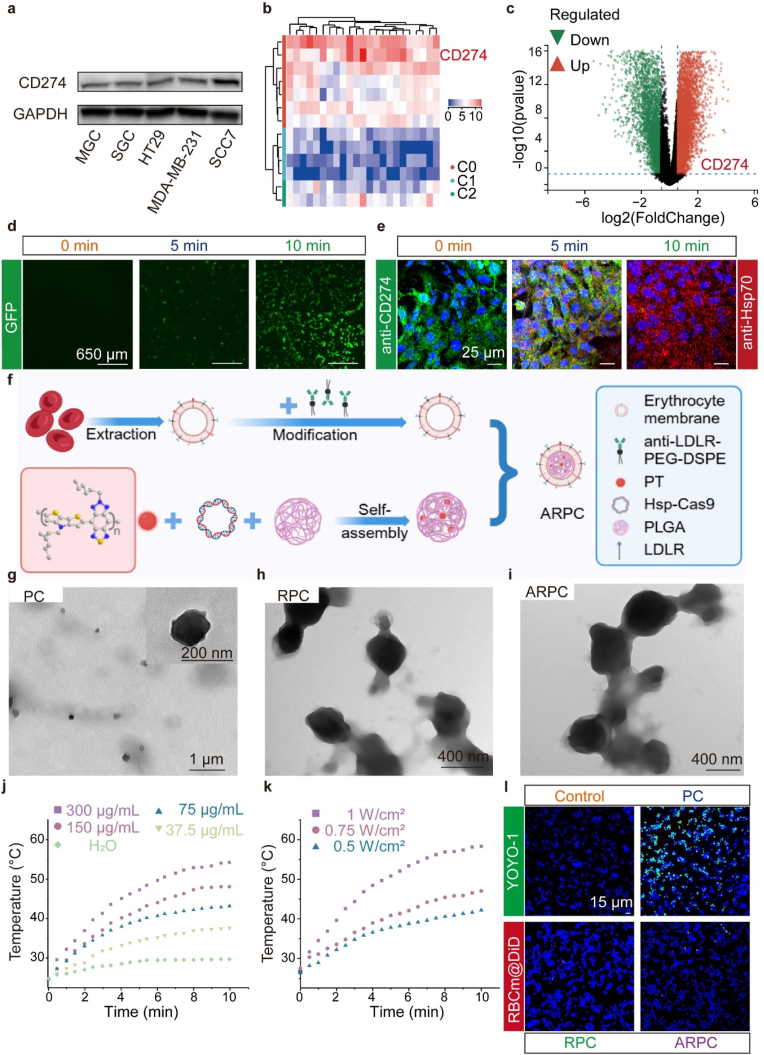
Preparation and characterization of ARPC. a) Utilize Western blot analysis to evaluate the expression of CD274 in MGC, SGC, HT29, MDA-MB-231, and SCC7 cell lines. b, c) Analyze genomic data from patients with HNSCC in TCGA database to assess the expression levels of upstream genes regulating CD274. d) Fluorescence microscopy of GFP protein expression in SCC7 cells following transfection with Lipofectamine 2000. e) The expression levels of Hsp70 and CD274 proteins in SCC7 cells after treatments, as assessed by fluorescence microscopy, Scale bar = 25 μm. f) Schematic illustration of the preparation of ARPC. g) TEM image of PC. h) TEM image of RPC. i) TEM image of ARPC. j) Photothermal conversion of ARPC with different concentration (1060 nm, 0.75 W/cm^2^) during 10 min laser irradiation in vitro. k) Photothermal conversion of ARPC with different power (1060 nm, 300 μg/mL) during 10 min laser irradiation in vitro. l) CLSM images in RAW264.7 cells after incubation with PC, RPC and ARPC. (Scale bar: 15 μm).

We analyzed expression data from 26 cancer types (Fig. S1) and confirmed CD274 expression in HNSCC by Western blotting (WB) (Fig. 1a). Using volcano plots and heatmaps, we identified CD274 as a key regulator of CD274 (Fig. 1b and c), guiding sgRNA design for the CRISPR/Cas9 system. To obtain specified promoter-directed CRISPR/Cas9 vector, we replaced the endogenous chicken β-actin promoter in pCP construct, thereby generating the desired Hsp-Cas9 plasmid (Tables S1 and S2). Furthermore, as shown in Fig. S2, we created a derivative Hsp-Cas9, enabling the simultaneous expression of Cas9 and green fluorescent protein (GFP). As shown in Fig. S3, to first assess whether the empty Hsp-Cas9 vector affects CD274 gene knockout, we transfected cells with the vector eSpCas9-2A-GFP(PX458) using Lipofectamine 2000, followed by heating for varying durations of 0, 5, 10, and 15 min. After heating, we performed agarose gel electrophoresis with T7 Endonuclease I to separate DNA fragments. The results showed no significant changes in fragment sizes. Similarly, cells were transfected with Hsp-Cas9 plasmids containing different sgRNAs using Lipofectamine 2000, followed by 10 min of heating. Notably in Fig. S4, sgRNA2 exhibited a more pronounced knockout effect compared to other sgRNAs. Consequently, for subsequent experiments, sgRNA2, demonstrating superior editing efficacy, will be utilized as the final therapeutic sgRNA. Optimal knockout conditions for sgRNA2 were determined by varying heating durations (Fig. 1d), with 10 min showing the highest GFP expression as confirmed by flow cytometry (Fig. S5). Each individual fluorescence channel during imaging is depicted in Fig. S6. Western blot analysis confirmed upregulation of Hsp70 and downregulation of CD274 with increased heating time, most notably at 10 min (Figs. S7 and 1e). Each individual fluorescence channel during imaging is depicted in Fig. S8. After optimizing Hsp-Cas9 and knockout conditions, we prepared nanoparticles as shown in Fig. 1f. The zeta potential decreased from −24 mV to −12.9 mV after α-LDLR-loaded RBCm encapsulation, indicating reduced negative charge (Fig. S9). Similarly, dynamic light scattering (DLS) was employed to characterize the particle sizes of PC, RPC, and ARPC (Fig. S10). The initial average particle size of PC was 135 nm, which increased to 232 nm for RPC after RBCm encapsulation, and ultimately, ARPC exhibited an average particle size of 313 nm. Transmission electron microscopy (TEM) confirmed PC's spherical shape at 150 nm (Fig. 1g). To verify the encapsulation of plasmid DNA and photothermal material PT, we performed scanning transmission electron microscopy (STEM) and energy dispersive spectroscopy (EDS) mapping. As shown in Fig. S11, the presence of phosphorus (P) is indicative of plasmid DNA, while sulphur (S) is characteristic of PT. The overlap of these elements with the high-angle annular dark field (HAADF) image confirms the incorporation of both plasmid DNA and photothermal material PT within PC nanoparticles. RBCm coating and α-LDLR incorporation on PC were visualized in Fig. 1h, with α-LDLR on RPC surface (Fig. 1i). Coomassie staining confirmed RBCm and α-LDLR presence in RPC and ARPC, indicating no protein loss (Fig. S12). To assess the presence of α-LDLR on the RBCm surface, we utilized DID staining and performed high-resolution confocal microscopy to visualize the colocalization of RBCm and α-LDLR. As shown in Fig. S13, α-LDLR (yellow) is located around RBCm (red), confirming the successful incorporation of α-LDLR as intended. Ultrasound had minimal impact on Hsp-Cas9 content (Fig. S14), and optimal Hsp-Cas9 loading was at a 4:1 ratio (Fig. S15). UV absorbance confirmed complete encapsulation of components in ARPC (Fig. S16). ARPC nanoparticles showed controllable photothermal effects in PBS under 1060 nm laser, adjustable by concentration (Fig. 1j) and power (Fig. 1k). They maintained photostability through laser cycles (Fig. S17). Optimal conditions were found at 0.75 W/cm^2^, 300 μg/mL for 10 min (Fig. S18), with a high photothermal conversion efficiency (PCE) of 39.32 % (Fig. S19). The UV–visible spectrum showed no shifts, indicating stability (Fig. S20). At 300 μg/mL, ARPC achieved 30 % PA intensity under laser irradiation (Fig. S21). To test immune evasion, PC, RPC, and ARPC were incubated with RAW264.7 macrophages. Confocal laser scanning microscopy (CLSM) images showed reduced fluorescence in RPC and ARPC-treated macrophages, suggesting RBCm coating enhanced immune evasion (Fig. 1l). Individual fluorescence channels are in Fig. S22. Flow cytometry confirmed a phagocytosis rate drop from 100 % for PC to 7.8 % for RBCm-encapsulated ARPC (Fig. S23). Furthermore, cellular uptake of ARPC by DC2.4 and RAW264.7 cells at different time points was analyzed by flow cytometry, as shown in Fig. S24. Overall, ARPC demonstrated efficient photothermal and photoacoustic performance alongside immune evasion from macrophages.

### 2.2 Cellular Uptake and Cytotoxicity Analysis

**Fig. 2:**
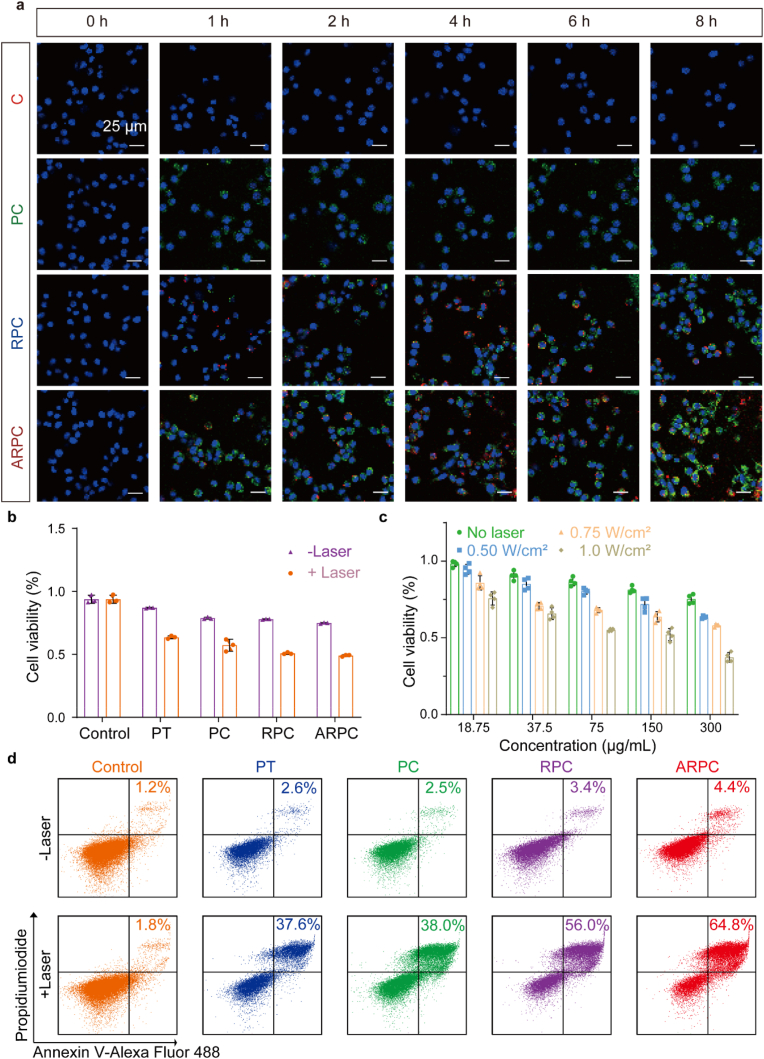
Cellular Uptake and Cytotoxicity Analysis. a) CLSM images of SCC7 cells incubated with C (CRISPR/Cas9@YOYO-1), PC (PC@YOYO-1), RPC (RPC@YOYO-1), ARPC (ARPC@YOYO-1) for different times. Green (YOYO-1), red (DID), (Scale bar: 25 μm). b) After treatment under various conditions, cell viability was assessed using the CCK-8 assay (1060 nm, 0.75 W/cm^2^, 5 min). Data are expressed as means ± SEM (n = 3). c) After treatment under various conditions, including different concentrations and groups, cell viability was evaluated using the CCK-8 assay (1060 nm, 0.75 W/cm^2^, 5 min). Data are presented as means ± SEM (n = 3). d) After subjecting different groups to laser irradiation (1060 nm, 0.75 W/cm^2^, 5 min), apoptosis was measured by flow cytometry. (For interpretation of the references to colour in this figure legend, the reader is referred to the Web version of this article.)

Next, we assessed the cellular uptake of C, PC, RPC, and ARPC in SCC7 cells, utilizing CRISPR/Cas9 with YOYO-1 to generate green fluorescence. Cellular uptake was analyzed by flow cytometry in the FITC channel (Fig. S25), showing ARPC, with α-LDLR receptors, had the highest internalization efficiency, supporting our design. Post-RBCm encapsulation, ARPC entered cells and underwent cleavage (Fig. 2a). The fluorescence intensity was quantified (Fig. S26), and CLSM showed ARPC's green fluorescence separating from endosomes and reaching the nucleus after 6 h, essential for genome editing (Fig. S27). Individual fluorescence channels are shown in Figures S28 and Figures S29. Cytotoxicity is vital for nanomaterial biomedicine applications. A CCK-8 assay on SCC7 cells showed lower cytotoxicity without laser exposure, but significant cell destruction under laser conditions (Fig. 2b). ARPC demonstrated increased cell killing with laser irradiation and concentration (Fig. 2c). Flow cytometry based apoptosis analysis revealed minimal variation in the -Laser group but a significant rise in apoptosis in the +Laser group (Fig. 2d). In the -Laser group, viable cells greatly outnumbered dead cells, with minimal apoptosis (Fig. S30). Individual fluorescence channels are shown in Fig. S31. Overall, ARPC showed the highest cytotoxicity under laser irradiation, likely due to specific cellular uptake.

### 2.3 Hsp70 promoter-driven genome editing of CD274 by ARPC

**Fig. 3:**
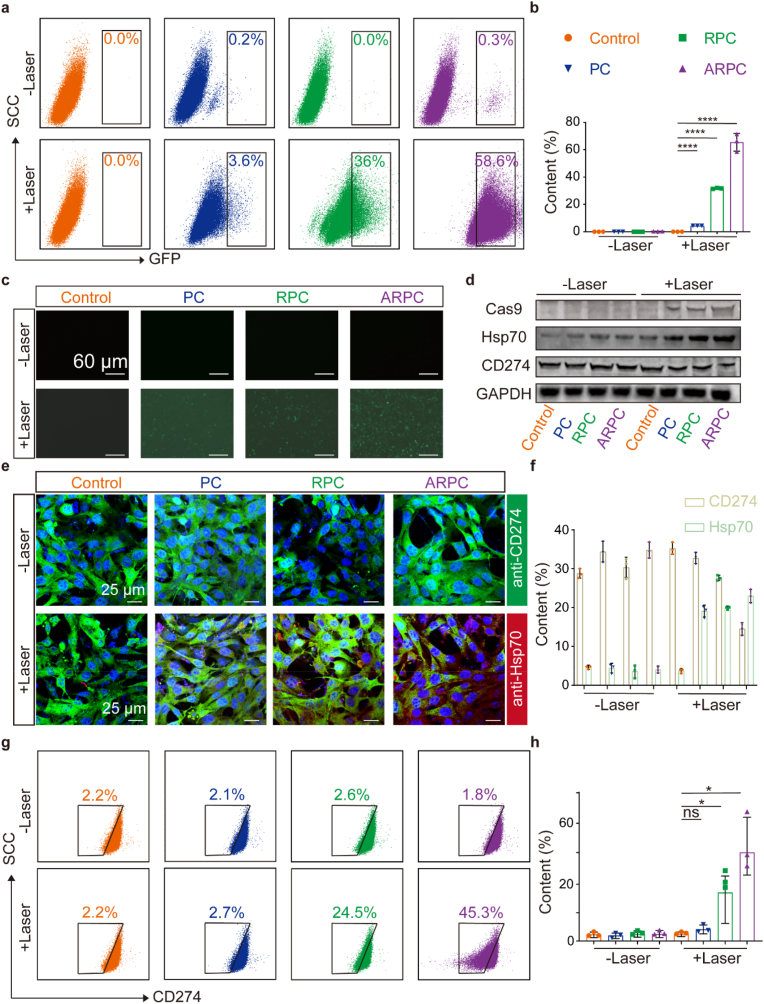
Hsp70 promoter-driven genome editing of CD274 by ARPC. a) GFP expression based on flow cytometry in SCC7 cells following transfection with PC, RPC, and ARPC after 0.75W/cm^2^ laser. b) Quantitative flow cytometry results for GFP expression. Mean ± S.D., n = 3. ∗P < 0.05; ∗∗P < 0.01; ∗∗∗P < 0.001; ∗∗∗∗P < 0.0001. c) Fluorescence microscopy image of the GFP in SCC7 cells. Scale bar: 60 μm. d) The expression levels of Hsp70 and CD274 proteins in SCC7 cells after treatments, as assessed by Western blot. e) The expression levels of Hsp70 and CD274 in SCC7 cells after treatments, as assessed by CLSM. Scale bar: 25 μm. f) Quantification of the fluorescence from anti-Hsp70 and anti-CD274 staining was performed using Image J. Mean ± S.D., n = 3. g) CD274 expression based on flow cytometry in SCC7 cells following transfection with PC, RPC, and ARPC after 0.75W/cm^2^ laser. h) Quantitative flow cytometry results for CD274 expression. Mean ± S.D., n = 3. ∗P < 0.05; ∗∗P < 0.01; ∗∗∗P < 0.001; ∗∗∗∗P < 0.0001.

In addition to cytotoxicity, ARPC nanocomplexes can efficiently deliver the Hsp70 promoter-driven CRISPR/Cas9 plasmid into SCC7 cells. Their photothermal conversion performance under a 1060 nm laser activates the Hsp70 promoter to express the Cas9 protein, which subsequently disrupts CD274 through CRISPR/Cas9-mediated gene editing. To validate our design, PC, RPC, and ARPC were co-cultured with SCC7 cells for 24 h and subsequently exposed to 1060 nm laser irradiation (0.75 W/cm^2^, 10 min). After an additional 48 h of incubation, the efficacy of the Hsp70 promoter-driven CRISPR/Cas9 system was assessed using flow cytometry and CLSM. As shown in Fig. 3a, in non-irradiated group (-Laser), GFP expression was low, remaining around 0.2 %. However, following laser irradiation, ARPC exhibited a GFP expression level of up to 58.6 %, significantly higher compared to PC (3.6 %) and RPC (36 %), indicating a marked improvement in transfection efficiency with ARPC. Fig. 3b displays the statistical analysis of the flow cytometry results. And Fig. 3c similarly demonstrates that ARPC treatment effectively enhances GFP expression in SCC7 cells. Each individual fluorescence channel during imaging is depicted in Fig. S32. Next, after co-culturing SCC7 cells with PC, RPC, or ARPC for 24 h, they were subjected to laser irradiation for 10 min, followed by an additional 72 h of incubation. WB and CLSM were then used to analyze the expression levels of various proteins, including Cas9, Hsp70, and CD274. As shown in Fig. 3d, following laser irradiation, Hsp70 expression increased progressively, while CD274 was downregulated and Cas9 was upregulated. In absence of laser irradiation, Cas9 expression was almost undetectable. To further corroborate these findings, SCC7 cells were treated with anti-Hsp70, anti-CD274, and anti-Cas9 antibodies and imaged using CLSM. Fig. 3e shows that with the upregulation of Hsp70 post-laser irradiation, there is a trend towards reduced CD274 expression. Each individual fluorescence channel during imaging is depicted in Fig. S33. Additionally, Fig. S34 indicates that Cas9 expression begins after laser irradiation. These analyzes collectively suggest that the upregulation of Hsp70 leads to the expression of Cas9, which in turn results in reduction of CD274 expression. Quantification of the fluorescence from anti-Hsp70 and anti-CD274 staining was performed using Image J, as shown in Fig. 3f. Finally, to assess the efficiency of CD274 knockout, flow cytometry was employed to detect CD274 expression in treated SCC7 cells. As shown in Fig. 3g, the proportion of CD274-negative cells in ARPC (+Laser) group reached 45.3 %, a significant increase compared to the 2.2 % observed in Control (+Laser) group. The statistical analysis of the flow cytometry results is shown in Fig. 3h. In summary, the ARPC-mediated CRISPR/Cas9 system demonstrates robust editing capabilities.

### 2.4 Whole-genome sequencing analysis of ARPC

**Fig. 4:**
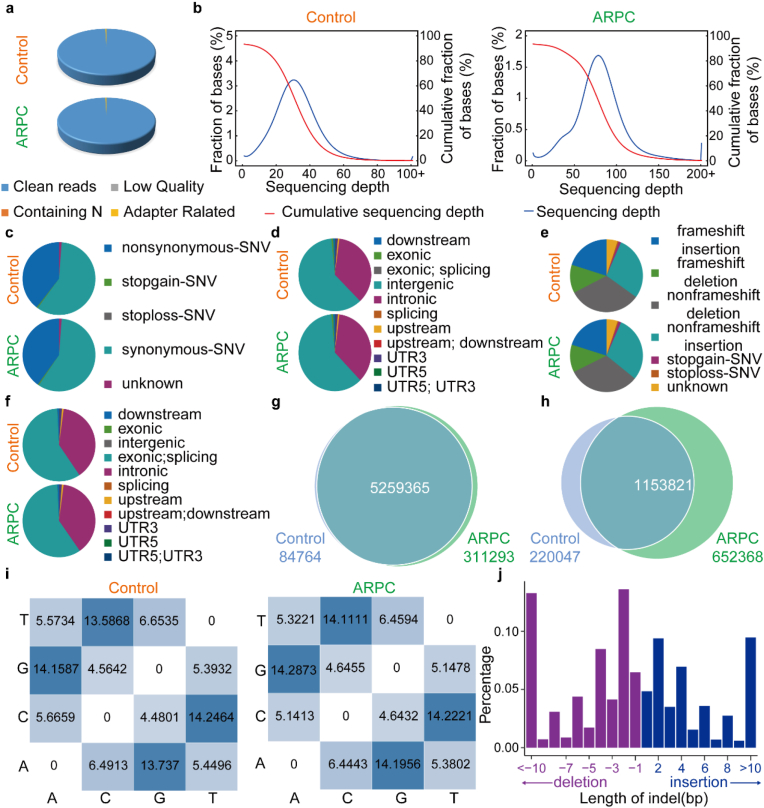
Whole-genome sequencing analysis of ARPC. a) Perform meticulous filtering of raw reads to obtain results of clean reads. b) Sequencing depth distribution maps for Control and ARPC. c) Number of SNPs across different genomic regions. d) Distribution of different types of SNPs across coding regions. e) Number of InDels across different genomic regions. f) Distribution of different types of InDels across coding regions. g) Shared and unique SNPs between Control and ARPC. h) Shared and unique InDels between Control and ARPC. i) Mutation type analysis of unique SNPs in Control and ARPC. j) Statistical analysis of insertion (insert) and deletion (deletion) lengths for unique InDels in Control and ARPC.

Genomic DNA was fragmented to 350 bp using Covaris sonicator, followed by end repair, A-tailing, and adapter ligation. Adapter details are in Table S3. Illumina sequencing required a Q30 ratio above 80 % and an error rate below 0.1 %. Quality control statistics are in Table S4, with filtered data proportions in Fig. 4a and Table S5. Quality scores and error rates are in Table S6, and base error rates in Figure S35 A, T, G, and C skew were assessed (Fig. S36). Sequencing data quality was above Q30, with base quality distribution in Fig. S37. The effective sequencing data were aligned to the reference genome using BWA, resulting in initial alignment in BAM format [43]. The alignment results were then sorted using SAM tools and duplicate reads were marked with Picard [44].Finally, we performed coverage and depth statistics on the alignment results with duplicates marked, as shown in Table S7. The sequencing depth distribution plots for each sample group derived from this analysis are illustrated in Fig. 4b. With further analysis delineating the number of SNPs across different genomic regions and coding regions, as detailed in Table S8, Table S9, Fig. 4c and d. It can be observed that the differences in SNP distribution between the Control and ARPC groups in exonic regions are primarily due to nonsynonymous and synonymous variants. The distribution of InDel numbers in various genomic regions and different types of InDels in coding regions is presented in Table S10, Table S11, Fig. 4e and f. When the number of samples was fewer than five, a Venn diagram was generated, with results as illustrated in Fig. 4g. Similarly, shared and unique InDels among different samples were analyzed. When the number of samples was fewer than five, a Venn diagram was generated, with results as illustrated in Fig. 4h [45]. It can be observed that the differences between the Control and ARPC groups, as determined by SNPs, are minimal, while the primary distinction relies on InDels. Subsequently, all mutation sites were compared, and unique SNP mutations in edited samples were identified [46]. The unique SNPs in each edited sample were then categorized by mutation type as shown in Fig. 4i. The unique InDels in each edited sample were then categorized by insertion (insert) and deletion (deletion) lengths, with results illustrated in Fig. 4j. The Cas-OFFinder software was utilized to compare sequences and predict off-target sites across the entire genome [47]. As illustrated in Table 1, these results are derived from the CD274 gene located on chromosome 19. Withinse sgRNA homologous regions, we screened for NGG motifs and visualized the results using a Circos plot, as shown in Fig. S38. This plot provides a comprehensive overview of the potential NGG off-target sites detected on the chromosomes or the off-target potential associated with different PAM sequences on the same chromosome.

**Table 1:** SNPs and InDels Variations in Homologous Region of sgRNA (Chrom: Chromosome of the variant; Pos: Position of the variant; Func: Region of the variant site; Ref: Reference sequence base; Alt: Base in detected sample.).

#CHROM		POS	Func	REF	ALT
19	SNPs	5543976	UTR3	A	G
		5543980	UTR3	C	T
		23044966	intergenic	C	T
		18613943	intronic	A	G
		45834171	intronic	C	T
1		135046967	exonic:unknown	A	G
3		104704293	exonic:nonsynonymous SNV	C	T

### 2.5 The intrinsic capability of ARPC to induce ICD

**Fig. 5:**
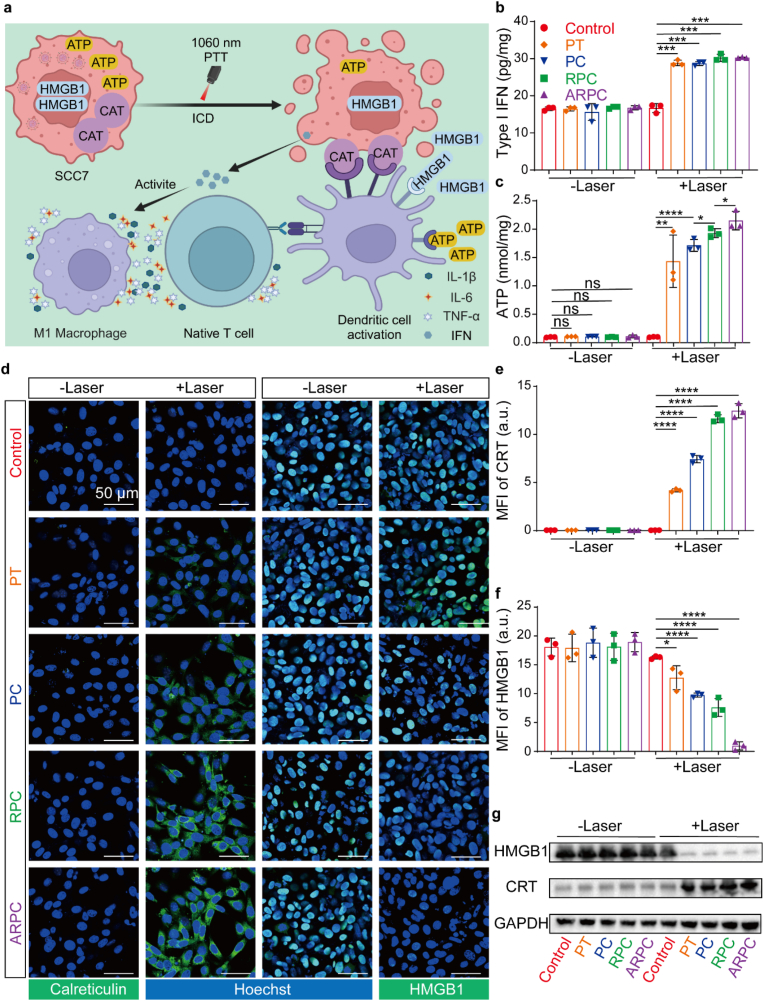
The intrinsic capability of ARPC to induce ICD. a) Schema of immunogenic cell death, and the cell surface expression and the release of such molecular patterns can stimulate tumor antigen presentation, thereby activating adaptive immunity. b) Quantitative results of Type I IFN were obtained using an ELISA kit. Mean ± S.D., n = 3. ∗P < 0.05; ∗∗P < 0.01; ∗∗∗P < 0.001; ∗∗∗∗P < 0.0001. c) Quantitative results of Type I IFN were obtained using an ATP assay kit. Mean ± S.D., n = 3. ∗P < 0.05; ∗∗P < 0.01; ∗∗∗P < 0.001; ∗∗∗∗P < 0.0001. d) Fluorescence microscopy image of the Calreticulin and HMGB1 in SCC7 cells. Scale bar: 50 μm. e) Quantification of the fluorescence from anti-Calreticulin staining was performed using Image J. Mean ± S.D., n = 3. ∗P < 0.05; ∗∗P < 0.01; ∗∗∗P < 0.001; ∗∗∗∗P < 0.0001. f) Quantification of the fluorescence from anti-HMGB1 staining was performed using Image J. Mean ± S.D., n = 3. ∗P < 0.05; ∗∗P < 0.01; ∗∗∗P < 0.001; ∗∗∗∗P < 0.0001. g) The expression levels of Calreticulin and HMGB1 proteins in SCC7 cells after treatments, as assessed by Western blot.

ICD transpires when SCC7 cells are subjected to specific stressors, leading to modifications in cell membrane and immune signaling within tumor microenvironment. During ICD, calreticulin (CRT) is translocated to the surface of tumor cells, high mobility group box 1 protein (HMGB1) and adenosine-5'-triphosphate (ATP) are secreted, and interferon (IFN) is synthesized by the tumor and immune cells [48,49]. As shown in Fig. 5a, these damage associated molecular patterns (DAMPs) can trigger the activation of CD8^+^ dendritic cells (DCs), which subsequently activate the adaptive immune system against tumor antigens. Initially, in Fig. 5b, the detection of IFN exhibits a comparable trend. The concentration increased from 16.8014 nmol/mg in Control (+Laser) group to 30.1937 nmol/mg in ARPC (+Laser) group. Similarly, ATP release assays were performed as shown in Fig. 5c. Following laser irradiation, ATP levels significantly increased, rising from 0.1 pg/mg in Control (+Laser) group to 2.03 pg/mg in ARPC (+Laser) group. Subsequently, the expression of the relevant proteins CRT and HMGB1 was analyzed using WB and CLSM. In Fig. 5d, CRT showed significant expression following laser irradiation, with the ARPC (+Laser) treatment demonstrating the most pronounced effect. Similarly, HMGB1 exhibited comparable results; with laser exposure, HMGB1 expressed in nucleus was secreted out of the cell, with the ARPC (+Laser) treatment showing the least nuclear HMGB1. Each individual fluorescence channel during imaging is depicted in Figure S39 and 40. Fig. 5e and f respectively present the quantitative results of the fluorescence intensity for anti-CRT and anti-HMGB1, based on ImageJ. In Fig. 5g, a similar conclusion is drawn. CRT expression progressively increases with laser irradiation, whereas HMGB1 expression gradually decreases.

### 2.6 In vitro induction of ICD-related immune cells

**Fig. 6:**
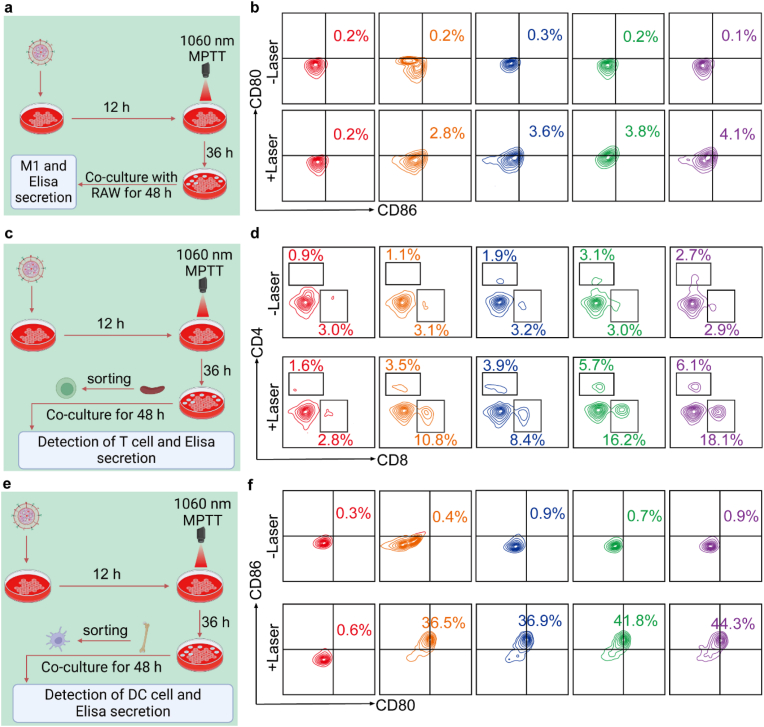
In vitro induction of ICD-related immune cells. a) Schematic representation of the co-culture experiment involving RAW and SCC7 cells. b) Representative flow cytometry images of M1 (CD80^+^, CD86^+^). c) Schematic representation of the co-culture experiment involving T cell and SCC7 cells. d) Representative flow cytometry images of CD8^+^ or CD4^+^. e) Representative flow cytometry images of mature dendritic cells (CD80^+^ and CD86^+^) gated on CD45^+^ and CD11c^+^ cells. f) Representative flow cytometry images of CD80^+^ and CD86^+^.

After confirming that PC, RPC, and ARPC can induce SCC7 cells to produce relevant immune factors, primary cells were extracted from mice to further simulate in vivo immune system activation through co-cultivation and induction. Fig. 6a illustrates the induction of M1 macrophages in RAW264.7 cells. After co-culturing PT, PC, RPC, and ARPC with SCC7 cells, the resulting supernatants were then used to co-culture with RAW264.7 cells. Flow cytometric analysis of treated RAW cells was performed using anti-CD80 and anti-CD86. The results, shown in Fig. 6b, reveal that double positive ARPC (-Laser) cells constitute only 0.2 %, while ARPC (+Laser) cells increase to 5.0 %, demonstrating a significant enhancement. This indicates that laser irradiation improves the ARPC's ability to induce M1 macrophage production compared to other groups. Overexpression of CD274 in tumor cells significantly impairs T cell proliferation and activation through its interaction with PD-1. To investigate whether the knockout of CD274 further increases the number of CD8^+^ T cells, primary T cells from mice were co-cultured using the same method, and flow cytometric analysis was performed with anti-CD4 and anti-CD8 (Fig. 6c and. d). Laser irradiation leads to a increase in CD4^+^ T cells and CD8^+^ T cells, reaching 6.1 % and 18.1 in ARPC(+Laser) group, compared to 2.7 % and 2.9 % in ARPC(-Laser) group. This substantial rise in CD8^+^ T cells may partly result from increased thermal damage caused by ARPC uptake, but more significantly, from the further activation of T cells due to the CD274 knockout. Induced ICD of tumor cells and the subsequent release of DAMPs facilitate the maturation of DCs. To assess whether these processes were occurring, SCC7 cells were pretreated with PT, PC, RPC, or ARPC, followed by exposure to 1060 nm laser irradiation, and then co-cultured with immature DCs derived from mouse bone marrow (Fig. 6e). After further incubation, the percentage of mature DCs (CD80^+^/CD86^+^) was quantified by flow cytometry. Fig. 6f shows that the percentages of mature DCs in ARPC (+Laser) and PT (+Laser) groups were 44.3 % and 36.5 %, respectively. In contrast, without 1060 nm laser irradiation, treatment with PT (0.4 %) or ARPC (0.9 %) had negligible effects on DC maturation compared to treatment with PBS (0.3 %). These findings collectively demonstrate that the ARPC component of the CRISPR/Cas9 system can induce ICD of SCC7 cells and subsequently enhance DC maturation under 1060 nm laser irradiation in vitro.

### 2.7 In vivo biodistribution and antitumor efficacy

**Fig. 7:**
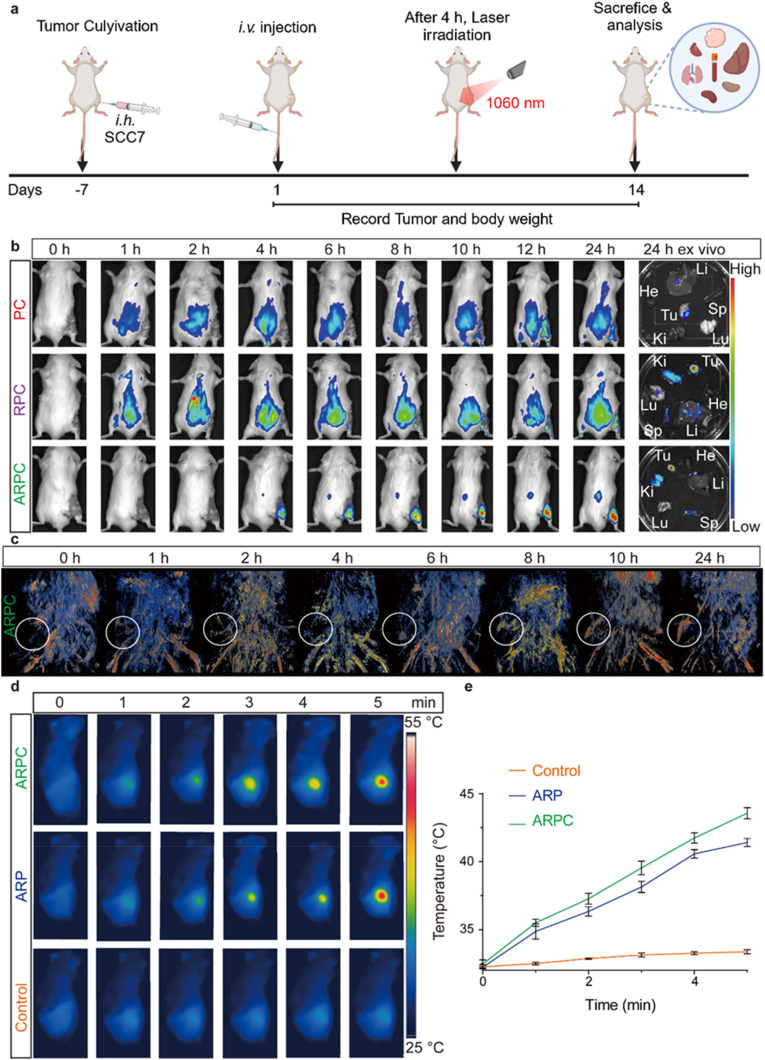
In vivo biodistribution and antitumor efficacy. a) Schematic diagram of treatment plan for subcutaneous SCC7 tumor-bearing mice. b) Fluorescence images of tumor sites at different times of PC, RPC and ARPC administration and in vitro fluorescence images of major organs and tumors of mice 24 h after injection. c) PA imaging in vivo of 24 h with tail vein injection of ARPC. d) Infrared thermograms of tumor in mice after injection of Control, ARP and ARPC through tail vein during 5 min irradiation. e) Temperature changes of tumor in mice with tail vein injection of Control, ARP and ARPC after 1060 nm laser irradiation during 5 min. All data represents means ± SEM (n = 3).

As previously mentioned, ARPC not only can be internalized by cells in vitro due to the presence of α-LDLR but also downregulates CD274, thereby enhancing the immune response induced by ICD. Fig. 7a illustrates the treatment protocol for subcutaneous tumor bearing mice, including tumor inoculation, treatment, and subsequent biochemical assays. To assess whether ARPC exhibits similar active targeting effects on tumor tissues in vivo, SCC7 cells were injected subcutaneously into mice. Once the tumors reached the desired volume, PC, RPC, and ARPC were administered via tail vein injection. Tumor targeting was then monitored using a fluorescence living image system. The results are shown in Fig. 7b. PC, as a simple nanoparticle, required approximately 10 h to passively accumulate at the tumor site, and exhibited relatively low fluorescence intensity. In contrast, RPC showed fluorescence at the tumor site within 4 h and demonstrated prolonged circulation within body, with continuous accumulation at the tumor site and increasing fluorescence up to 24 h. Finally, ARPC, which is coated with α-LDLR, exhibited significantly stronger fluorescence at 4 h compared to the previous two groups and continued to accumulate at the tumor site, demonstrating the most effective targeting of tumor tissues. Fluorescence quantification is shown in Fig. S41. These findings were further supported by photoacoustic imaging (PAI) (Fig. 7c and Fig. S42). Furthermore, to achieve MPTT, the temperature was monitored using an infrared thermal imaging camera. Fig. 7d and e present the measurement of tumor temperature during treatment for three groups: Control, ARP, and ARPC. Under the condition of 0.75 W/cm^2^ and a 5 min irradiation, both ARP and ARPC were able to reach the 42 °C required for MPTT.

### 2.8 Subcutaneous tumor treatment and CD274 downregulation effects

**Fig. 8:**
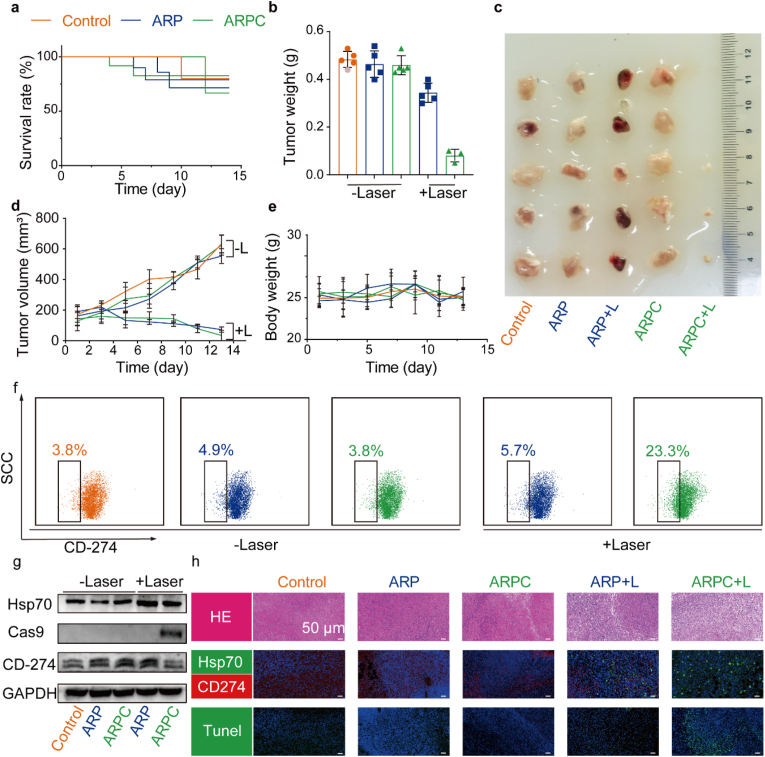
Subcutaneous tumor treatment and CD274 downregulation effects. a) Survival curves of tumor-bearing mice during the treatment process. b) Tumor weight change curve of mice after treatment irradiation. All data represents means ± SEM (n = 5). c) Images of tumor when therapy was over. d) Tumor volume change curve of mice after treatment irradiation. All data represents means ± SEM (n = 5). e) Body weight change curve of mice after treatment irradiation. All data represents means ± SEM (n = 5). f) Flow cytometry analysis of CD274 expression in cancer cells at the conclusion of anticancer therapy. g) The expression of CD274, Cas9, and Hsp70 protein in tumor tissue examined by Western blotting. h) Representative immunofluorescence images of terminal deoxynucleotidyl transferase dUTP nick end labeling (TUNEL) staining, CD274, and Hsp70 in tumor tissue.

After the treatment, the number of mouse deaths during the treatment process was first tallied, and survival curves were plotted as shown in Fig. 8a. The curves indicate that there was little difference in survival rates before and after treatment. Tumor tissues were then dissected, and tumor weights were recorded, as illustrated in Fig. 8b. Additionally, photographs of the tumor tissues were taken for each group, as shown in Fig. 8c. It is evident that the presence or absence of laser treatment has a significant impact on tumor growth, with the smallest tumors observed in ARPC + L group, suggesting that ARPC may have an enhanced immune-stimulating effect compared to ARP + L. We used Propidium iodide (PI) to stain the single cell suspension prepared from the treated tumor tissue, and analyzed it with the lost cell analyzer. PI can't cross the cell membrane of living cells, but can only cross the disordered area of dead cell membrane and reach the nucleus, and it embeds into the DNA double helix of cells to produce red fluorescence (Ex = 535 nm, Em = 617 nm), so PI only dyes dead cells. Results As shown in Fig. S43, we can see that the positive rate of PI staining in the Control group is the lowest, while the mortality rates in the ARP + L and ARPC + L groups are only 13.44 % and 15.21 %. This shows that the survival rate of tumor cells after irradiation is high, which achieves a mild photothermal effect, and also provides conditions for CD274 knockout of CRISPR/Cas9. Fig. 8d depicts the changes in tumor volume throughout the treatment process. Fig. 8e shows the changes in mouse body weight, revealing that there was minimal variation in body weight, which remained stable at approximately 25 g. Next, the downregulation of CD274 in tumor tissues was analyzed. In Fig. 8f, flow cytometry was first used to examine tumor cells treated with α-LDLR. It was observed that the ARPC + L group exhibited the highest rate of CD274 negativity, approximately four times higher than other groups. To investigate whether the downregulation of CD274 was associated with Hsp70 activation, WB was performed on tumor tissues post-treatment. As shown in Fig. 8g, both ARP + L and ARPC + L treatments induced Hsp70 upregulation due to photothermal therapy, only the Cas9 of ARPC + L group is increasing, and so the CD274 expression was notably lower in ARPC + L group. Immunofluorescence staining of tumor tissue sections with anti-Hsp70 and anti-CD274 antibodies, as depicted in Fig. 8h, confirmed these findings, illustrating the precise editing function of the ARPC nanoplatform in Hsp70 induction. Additionally, HE and TUNEL staining of tumor cells were conducted, and the results were as expected, with ARP + L and ARPC + L groups showing stronger green fluorescence in TUNEL staining. This further elucidates the therapeutic efficacy of the tumor treatment.

### 2.9 The immunogenic cell death effect in tumor sites

**Fig. 9:**
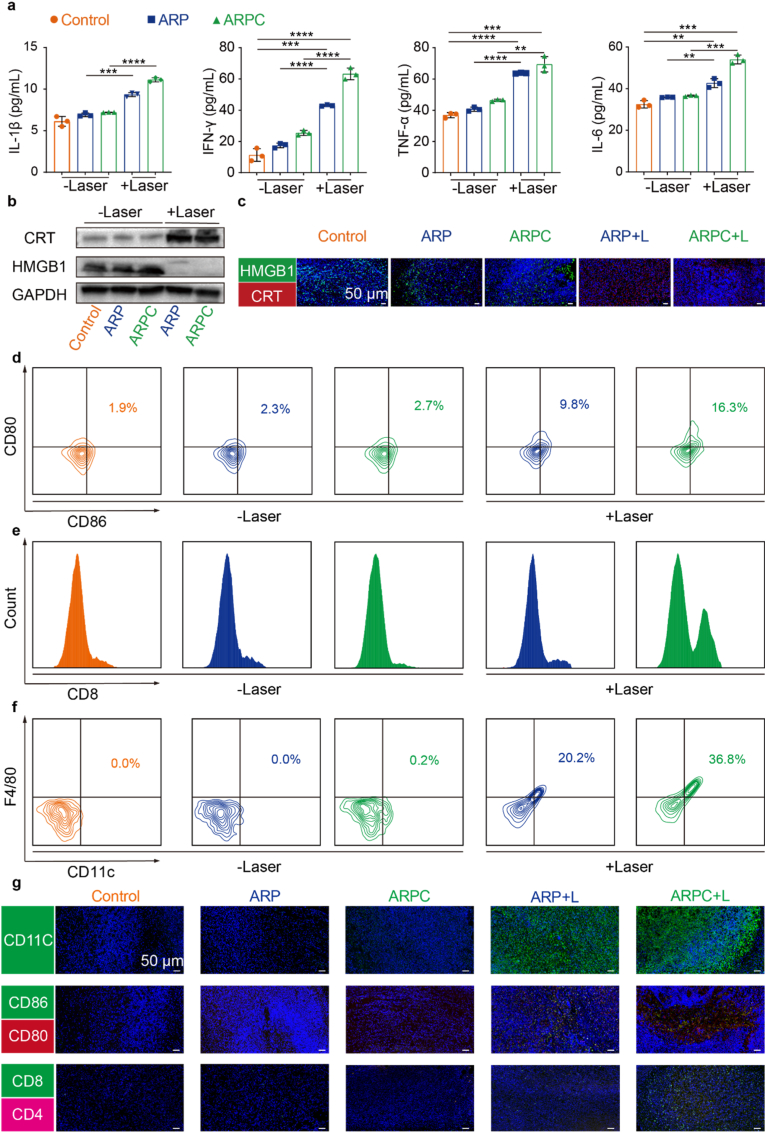
The immunogenic cell death effect in tumor sites. a) ELISA analysis was performed to assess the levels of IFN-γ, TNF-α, IL-6, and IL-1β in tumor tissue following various treatments. Mean ± S.D., n = 3. ∗P < 0.05; ∗∗P < 0.01; ∗∗∗P < 0.001; ∗∗∗∗P < 0.0001. b) Western blot analysis was conducted to evaluate the protein expression of CRT and HMGB1 in SCC7 tumor tissue. c) Representative immunofluorescence images of CRT and HMGB1 in tumor tissue. d) Representative flow cytometry images illustrating the percentage of mature dendritic cells (CD80^+^, CD86^+^ cells within CD45^+^, CD11c + cells) in tumor. e) Representative flow cytometry images and quantitative statistical analysis of the CD8^+^ T cell percentage (within CD3^+^ cells). f) Representative flow cytometry images of the macrophage percentage (CD11c^+^, F4/80^+^ cells). g) Representative immunofluorescence images of CD11c, CD86, CD80, CD8 and CD4 in tumor tissue.

**Fig. 10:**
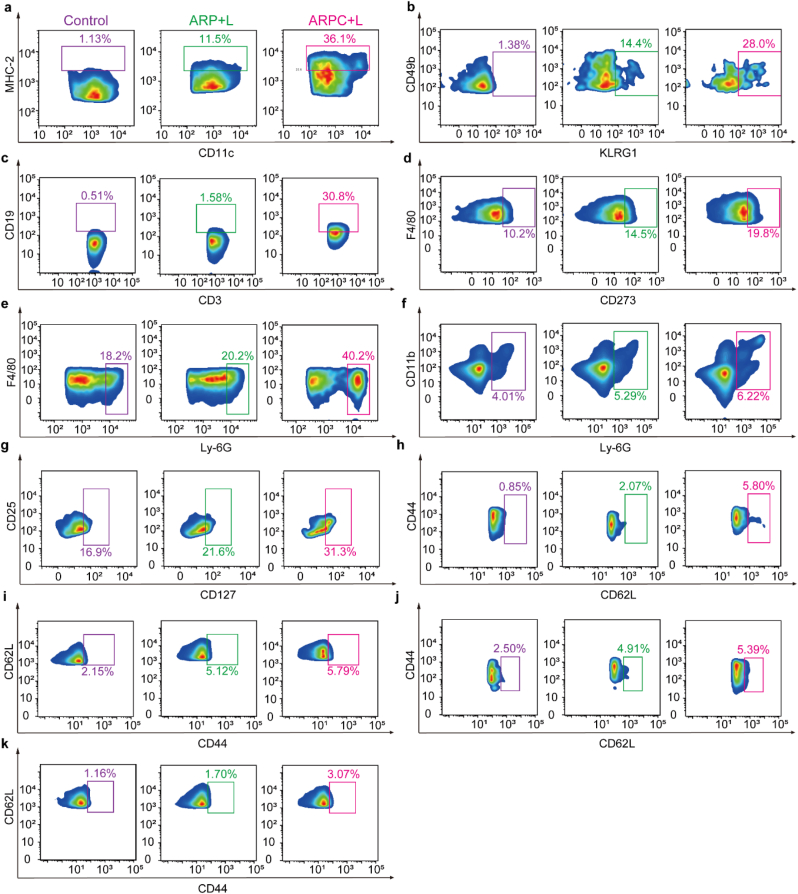
Analyzing the immune cells within the tumor immune microenvironment of relevant groups after treatment using mass cytometry. a) Dendritic cells. b) Natural killer cell (KLRG1+). c) B cell. d) Macrophage (PD-L2+) e) Neutrophils. f) Monocytes. g) Regulatory T cells. h) Naive CD4^+^ T cells. i) Effector memory CD4^+^ T cells. j) Navie CD8^+^ T cells. k) Effector memory CD8^+^ T cells.

To elucidate the mechanism underlying the promising antitumor efficacy induced by the Hsp70-promoter-driven CRISPR/Cas9 system, we first analyzed the relevant inflammatory cytokines associated with ICD. As shown in Fig. 9a, the levels of IL-1β, IFN-γ, TNF-α, and IL-6 were elevated following laser irradiation. For ICD associated proteins, CRT and HMGB1, we conducted Western blotting and immunohistochemical staining of tissue sections. In Fig. 9b, CRT was observed to upregulate after laser irradiation, whereas HMGB1 exhibited a decrease in expression. A similar pattern was observed in Fig. 9c. These results indicate that laser irradiation successfully induced ICD. Subsequently, we evaluated the response of immune cells in lymph nodes and tumors using multiparameter flow cytometry. We prioritized the investigation of mature DCs in tumor tissue (Fig. 9d). Compared to the Control group (1.9 %), the ARP (2.3 %) and ARPC (2.7 %) groups did not exhibit significant changes in percentages of CD80^+^ CD86^+^ DCs. However, the ARP + L and ARPC + L groups, which both demonstrated an induced ICD effect in tumor tissue, showed a dramatic increase in frequencies of mature DCs (9.8 % and 16.3 %, respectively). Furthermore, CD274 disruption blocks the PD-1/PD-L1 interaction, thereby enhancing the T cell mediated antitumor immune response. Consequently, tumor infiltrating lymphocytes (TILs) were further evaluated using multiparameter flow cytometry. As depicted in Fig. 9e, low levels of CD8^+^ T cell infiltration (as a percentage of CD3^+^ cells) were observed in tumors from mice treated with Control (1.7 %), ARP (1.3 %), and ARPC (1.0 %). Conversely, ARP + L treatment (5.2 %) and ARPC + L treatment (32.0 %) resulted in a modest enhancement of CD8^+^ T cell infiltration, indicating that the integration of genomic CD274 disruption and ICD induction effectively elevated the proportion of tumor infiltrating CD8^+^ T lymphocytes. Another indicator of the immune response is the increase in macrophages. To confirm this, tumor tissues were analyzed using flow cytometry with anti-F4/80 and CD11c staining. As shown in Fig. 9f, the ARPC + L group exhibited the highest ratio (36.8 %), followed by the ARP + L group (20.2 %), while the increases in remaining non-laser treated groups were not significant. Furthermore, tumor tissue sections were reanalyzed using immunofluorescence techniques. As depicted in Fig. 9g, the ARPC + L and ARP + L groups exhibited significantly higher indicators compared to the others. In order to analyze the changes in immune cells within the tumor immune microenvironment, we further analyzed the relevant groups after treatment using mass cytometry, and the results are shown in Fig. 10. In summary, tumor tissues were infiltrated by immune cells during treatment, and the CRISPR/Cas9 system-mediated downregulation of CD274 further activated T cells.

### 2.10 Inflammatory cells in lymph nodes and spleen

**Fig. 11:**
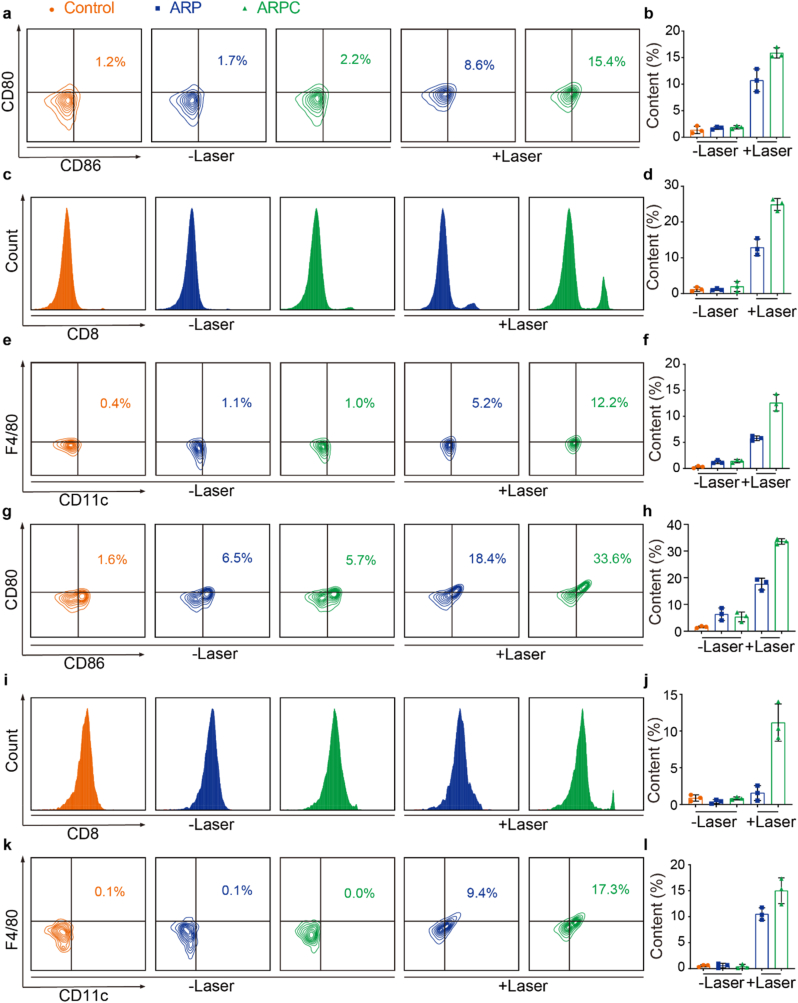
Inflammatory cells in lymph nodes and spleen. a) Representative flow cytometry images illustrating the percentage of mature dendritic cells (CD80^+^, CD86^+^ cells within CD45^+^, CD11c^+^ cells) in lymph nodes. b) Quantitative analysis of CD80^+^ and CD86^+^ cells based on flow cytometry in lymph nodes. Mean ± S.D., n = 3. ∗P < 0.05; ∗∗P < 0.01; ∗∗∗P < 0.001; ∗∗∗∗P < 0.0001. c) Representative flow cytometry images and quantitative statistical analysis of the CD8^+^ T cell percentage (within CD3^+^ cells) in lymph nodes. d) Quantitative analysis of CD8^+^ and CD4^+^ cells based on flow cytometry in lymph nodes. Mean ± S.D., n = 3. ∗P < 0.05; ∗∗P < 0.01; ∗∗∗P < 0.001; ∗∗∗∗P < 0.0001. e) Representative flow cytometry images of the macrophage percentage (CD11c^+^, F4/80^+^ cells) in lymph nodes. f) Quantitative analysis of CD11c^+^ and F4/80^+^ cells based on flow cytometry in lymph nodes. Mean ± S.D., n = 3. ∗P < 0.05; ∗∗P < 0.01; ∗∗∗P < 0.001; ∗∗∗∗P < 0.0001. g) Representative flow cytometry images illustrating the percentage of mature dendritic cells (CD80^+^, CD86^+^ cells within CD45^+^, CD11c^+^ cells) in spleen. h) Quantitative analysis of CD80^+^ and CD86^+^ cells based on flow cytometry in spleen. Mean ± S.D., n = 3. ∗P < 0.05; ∗∗P < 0.01; ∗∗∗P < 0.001; ∗∗∗∗P < 0.0001. i) Representative flow cytometry images and quantitative statistical analysis of the CD8^+^ T cell percentage (within CD3^+^ cells) in spleen. j) Quantitative analysis of CD8^+^ and CD4^+^ cells based on flow cytometry in spleen. Mean ± S.D., n = 3. ∗P < 0.05; ∗∗P < 0.01; ∗∗∗P < 0.001; ∗∗∗∗P < 0.0001. k) Representative flow cytometry images of the macrophage percentage (CD11c^+^, F4/80^+^ cells) in spleen. l) Quantitative analysis of CD11c^+^ and F4/80^+^ cells based on flow cytometry in spleen. Mean ± S.D., n = 3. ∗P < 0.05; ∗∗P < 0.01; ∗∗∗P < 0.001; ∗∗∗∗P < 0.0001.

Similarly, to fully evaluate and verify the activation of related cells, we used multi-parameter flow cytometry to evaluate the immune cell response in lymph nodes. Considering that ICD might induce DC maturation, we focused on investigating mature DC cells in lymph nodes. As shown in Fig. 11a, compared to the control group (1.2 %), the percentage of CD80^+^ and CD86^+^ DC in ARP group (1.7 %) and ARPC group (2.2 %) has no significant changes. In contrast, both ARP + L and ARPC + L groups showed induced ICD effect in lymph nodes, which showed that the frequencies of mature dendritic cells increased significantly (8.6 % and 15.4 % respectively). Fig. 11b presents the quantitative analysis of flow cytometry. Subsequently, T cells were analyzed. In Fig. 11c, the percentage of CD8^+^ T cells significantly increased in ARP + L and ARPC + L groups following laser irradiation, reaching 11.3 % and 23.6 %, respectively, compared to the other groups. Fig. 11d displays the quantitative analysis of flow cytometry. Finally, macrophages in lymph nodes were analyzed. As shown in Fig. 11e, the ARP + L and ARPC + L groups exhibited the highest proportions of macrophages compared to the other three groups, with values of 5.2 % and 12.2 %, respectively. Fig. 11f presents the quantitative analysis of macrophage increment. Next, a similar analysis was conducted on relevant cells in spleen. Fig. 11g shows the selection of mature DCs, with the ARP + L group (18.4 %) and the ARPC + L group (33.6 %) exhibiting significantly higher proportions compared to the other groups. Fig. 11h provides the statistical results following the selection of mature DC cells. Fig. 11i illustrates the analysis of CD8^+^ T cells, revealing a significant increase in proportion following laser irradiation. Fig. 11j presents the statistical analysis of the CD8^+^ and CD4^+^ cell ratios across different groups. Finally, Fig. 11k analyzes the quantity of macrophages, showing a substantial increase in ARP + L and ARPC + L groups, as anticipated. Fig. 11l provides the statistical results following flow cytometry sorting. In summary, the ICD-induced immune response is evident not only at the tumor site but also in spleen and lymph nodes, with similar conclusions drawn.

### 2.11 SCC7 in situ cancer and the targeting specificity of ARPC

**Fig. 12:**
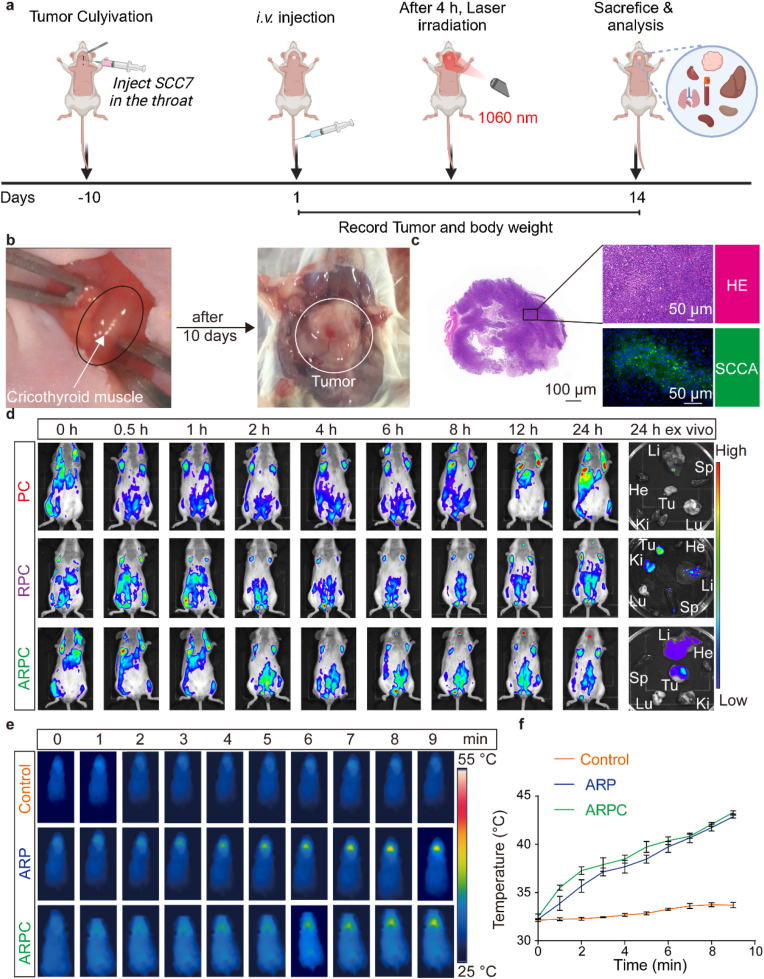
SCC7 in situ cancer and the targeting specificity of ARPC. a) Schematic of the treatment regimen for SCC7 tumor bearing mice in situ. b) Schematic of the construction of SCC7 laryngeal cancer. c) HE and SCCA immunostaining of tumor tissue sections. d) Fluorescence images of the tumor sites at different time points for PC, RPC, and ARPC, and ex vivo fluorescence images of major organs and tumors 24 h post-injection. e) Infrared thermographic images of tumors in mice following tail vein injection of control, ARP, and ARPC, during 9 min of irradiation. f) Temperature changes in tumors of mice injected with control, ARP, and ARPC after 9 min of 1060 nm laser irradiation. All data represents means ± SEM (n = 3).

ARPC demonstrates robust active targeting in subcutaneous SCC7 tumors and effective ICD induction through CD274 downregulation. To further validate its efficacy in SCC7 in situ cancer, we surgically established laryngeal cancer derived from SCC7 and administered PBS, ARP, and ARPC with laser irradiation via tail vein injection after 10 days (Fig. 12a). As shown in Fig. 12b, SCC7 cell suspensions injected into the cricothyroid muscle developed tumor tissue within 10 days. Subsequent HE and anti-SCCA staining of the tumor tissue are depicted in Fig. 12c. SCCA, a common antigen in head and neck squamous cell carcinoma patients, confirms the successful establishment of SCC7 laryngeal cancer [[50], [51], [52]]. The targeting specificity of ARPC in SCC7 laryngeal cancer was then investigated, with results shown in Fig. 12d. PC did not reach the tumor region within 24 h, whereas RPC and ARPC exhibited superior targeting, with ARPC showing a strong fluorescent signal within 6 h and continuous accumulation over 24 h. Fluorescence quantification is shown in Fig. S44. Finally, the achievement of the optimal MPTT temperature (42 °C) in SCC7 laryngeal cancer was observed. As shown in Fig. 12e, under 0.75 W/cm^2^ laser irradiation, in situ cancer required a longer duration to reach the target temperature compared to subcutaneous tumors, reaching approximately 42 °C only after 9 min. This delay may be due to the attenuation of NIR-II power with tissue depth. Fig. 12f presents the quantitative temperature results obtained using a thermal imaging camera. Overall, ARPC demonstrates targeting capability in in situ cancer as well.

### 2.12 Treatment efficacy and biocompatibility in SCC7 in situ cancer

**Fig. 13:**
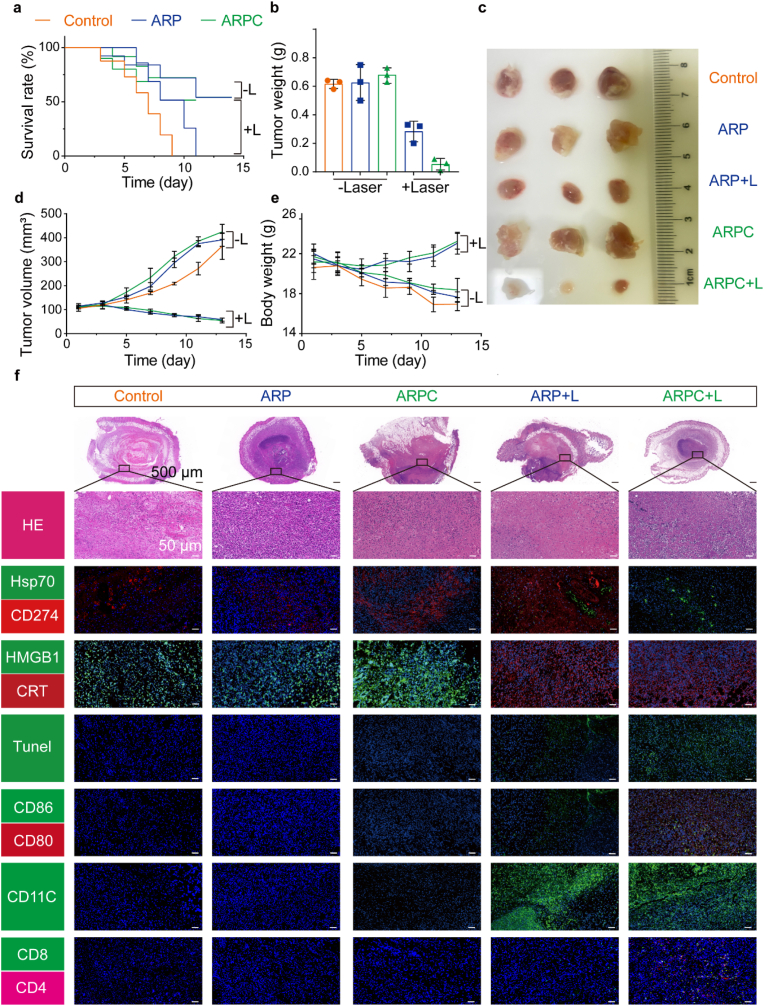
Treatment efficacy in SCC7 in situ cancer. a) Survival curves of tumor-bearing mice during the treatment process. b) Tumor weight change curve of mice after treatment irradiation. All data represents means ± SEM (n = 3). c) Images of tumor when therapy was over. d) Tumor volume change curve of mice after treatment irradiation. All data represents means ± SEM (n = 3). e) Body weight change curve of mice after treatment irradiation. All data represents means ± SEM (n = 3). f) Representative immunofluorescence images of terminal deoxynucleotidyl transferase dUTP nick end labelling (TUNEL) staining, CD274, Hsp70, HMGB1, CRT, CD80, CD86, CD11c, CD8, and CD4 in tumor tissue.

**Fig. 14:**
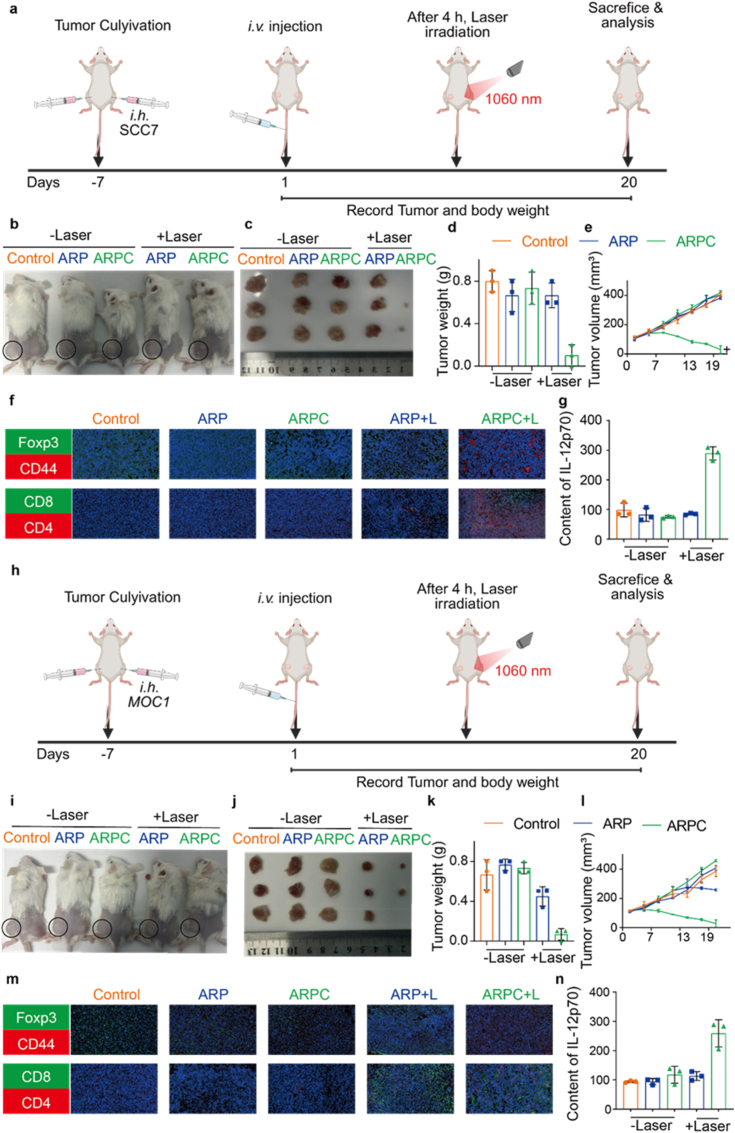
Treatment efficacy in the tumor distant model on Balb/c mice of SCC7 and MOC1 cell. a) Schematic of the treatment regimen for the tumor distant model on Balb/c mice of SCC7. b) Images of mice when therapy was over. c) Images of tumor when therapy was over. d) Tumor weight change curve of mice after treatment irradiation. All data represents means ± SEM (n = 3). e) Tumor volume change curve of mice after treatment irradiation. All data represents means ± SEM (n = 3). f) Representative immunofluorescence images of Foxp3, CD44, CD8 and CD4. g) ELISA analysis was performed to assess the levels of IL-12p70. h) Schematic of the treatment regimen for the tumor distant model on Balb/c mice of SCC7. i) Images of mice when therapy was over. j) Images of tumor when therapy was over. k) Tumor weight change curve of mice after treatment irradiation. All data represents means ± SEM (n = 3). l) Tumor volume change curve of mice after treatment irradiation. All data represents means ± SEM (n = 3). m) Representative immunofluorescence images of Foxp3, CD44, CD8 and CD4. n) ELISA analysis was performed to assess the levels of IL-12p70.

At the conclusion of the treatment cycle, to evaluate the therapeutic efficacy in mice, we first plotted survival curves. As shown in Fig. 13a, the survival rate of mice without laser irradiation was significantly lower compared to other groups. We attribute this to the laryngeal tumor affecting the mice's ability to eat, thereby impacting survival rates. Subsequently, we measured the tumor tissues post-treatment (Fig. 13b) and photographed them (Fig. 13c), demonstrating that ARPC + L exhibited superior therapeutic efficacy compared to other groups. We also collected data and plotted the changes in tumor volume during the treatment period, as shown in Fig. 13d. The tumor volumes in treatment groups ARP + L and ARPC + L exhibited a decreasing trend on day five and continued to decline throughout the treatment cycle. Fig. 13e presents the weight statistics of the mice. Due to the mice's inability to eat, the body weight in three control groups notably decreased to approximately 18 g. In contrast, the treatment groups ARP + L and ARPC + L maintained relatively stable body weights, around 22 g. We then performed section staining on the tumor tissues obtained through dissection, with results illustrated in Fig. 13f. Initially, for CD274 and Hsp70, we observed that Hsp70 expression was upregulated following light exposure, while CD274 exhibited a downregulation trend in ARPC + L group compared to the ARP + L group. This further confirms the successful editing of CD274 expression by CRISPR/Cas9 mediated through Hsp70. Regarding ICD related proteins CRT and HMGB1, CRT expression increased following laser irradiation, while HMGB1 decreased, indicating the occurrence of ICD. Additionally, TUNEL staining revealed further increased apoptosis in cells post-laser exposure. For assessing ICD-induced immune cell maturation, we employed anti-CD86, anti-CD80, anti-CD11c, anti-CD4, and anti-CD8 for section staining. Similarly, following laser irradiation, there was an increase in CD80^+^, CD86^+^, CD11c^+^, CD4^+^ and CD8^+^ expressions. These results confirm the successful occurrence of ICD and its capacity to induce the maturation of relevant immune cells. This also demonstrates the success of ARPC in treatment of SCC7 orthotopic tumors. To further analyze the antigen specific T cells generated in mice after treatment, we selected the SCC7 and MOC1 cell lines to establish a tumor distant model on Balb/c mice. As shown in Fig. 14, the T cells generated after laser irradiation in the ARPC group could reduce the volume and weight of the tumor. While evaluating the excellent therapeutic efficacy of ARPC, its biocompatibility cannot be overlooked. Therefore, HE staining was performed on the major organs of mice (heart, liver, spleen, lung, and kidney) post-treatment, as shown in Fig. S45. HE staining revealed no significant lesions in ARP + L and ARPC + L treatment groups. Subsequently, serum analysis of liver and kidney function markers (ALT, AST, BUN, and CREA) was conducted. As depicted in Fig. S46, all markers were within normal range. Additionally, erythrocyte sedimentation rate analysis was performed on whole blood. Fig. S47 shows that there was no significant erythrocyte sedimentation at 30 min. Finally, complete blood count parameters, including WBC, HGB, PLT, and LYM, were analyzed. The results, shown in Fig. S48, indicated that values were consistent across groups and within normal range. Due to ARP + L and ARPC + L inducing verification responses, they were not analyzed further. In summary, ARPC demonstrated no apparent biological toxicity during the treatment of tumor-bearing mice.

## 3 Conclusion

In summary, an NIR-II optogenetic CRISPR/Cas9 nanoplatform termed ARPC, has been successfully developed to address the ICB-related issues encountered in current HNSCC clinical treatments. This system incorporates α-LDLR to actively target SCC7 cells within hypoxic environments, while RBCm confers resistance to immune system clearance and extended circulation within bloodstream. The NIR-II activated Hsp70 promoter, leading to permanent genomic disruption of CD274, and thereby preventing immune escape by tumor cells. The ICD induction in SCC7 cells triggers a multifaceted anti-cancer immune response, including the promotion of DC maturation, an increased M1 macrophage ratio, enhanced CD8^+^ T cell infiltration and proliferation, and a reduction in proportion of immunosuppressive cells, thereby remodeling the tumor immune microenvironment. Notably, the ARPC system not only achieves efficient transfection through α-LDLR modified RBCm but also utilizes NIR-II optogenetic CRISPR/Cas9 specific promoter activation to induce spatiotemporally controlled gene editing, demonstrating significant potential for avoiding severe immune-related adverse events in clinical applications.

Furthermore, although the whole-genome sequencing results provided convincing biosafety evidence of short term of ARPC mediated CD274 gene editing, the effects of long term and durability of this therapeutic approach remain to be fully characterized. Future studies should include prolonged follow-up of cured mice to assess the persistence of CD274 disruption and detect any potential off-target modifications in major organs. In addition, continuous immunological monitoring will be necessary to identify possible autoimmune manifestations and to determine whether CD274 protein may be expressed again in recurrent tumor tissues. Such investigations will enable a more comprehensive evaluation of the safety of long term and stability of this NIR-II optogenetic CRISPR/Cas9 therapeutic system. In terms of clinical translation, while this study demonstrates the effective use of NIR-II for precise optogenetic activation in both subcutaneous and orthotopic HNSCC models, extending this approach to head and neck malignancies in deep, such as hypopharyngeal carcinoma, presents further challenges. The limited penetration depth of NIR-II irradiation in dense tissues could restrict its applicability in deep tumor regions. To overcome these limitations, future work may explore clinically feasible delivery strategies, including assistance with endoscope or fiber-optic laser irradiation, as well as the incorporation of guided by light probes or photothermal sensitizers to enhance energy deposition at target sites. These improvements would facilitate the adaptation of NIR-II optogenetic gene editing to complex anatomical locations and strengthen its translational potential for clinical oncology applications. Overall, this research presents an innovative alternative strategy for effective ICB treatment of HNSCC and offers hope for future tumor gene editing therapies.

## CRediT authorship contribution statement

**Yang Chen:** Writing – review & editing, Writing – original draft, Visualization, Validation, Software, Project administration, Investigation, Formal analysis, Data curation, Conceptualization. **Longcai Liu:** Validation, Resources, Investigation, Formal analysis, Data curation, Conceptualization. **Xiaojuan Hu:** Visualization, Validation, Methodology, Formal analysis, Data curation. **Yilin Huang:** Visualization, Resources, Methodology, Investigation. **Shijie Yao:** Software, Project administration, Funding acquisition. **Lichen Ji:** Software, Investigation. **Hai Zou:** Writing – review & editing, Supervision, Project administration, Funding acquisition. **Xiaozhou Mou:** Writing – review & editing, Supervision, Project administration, Funding acquisition, Conceptualization. **Yu Cai:** Writing – review & editing, Supervision, Resources, Project administration, Investigation, Funding acquisition, Conceptualization.

## Declaration of competing interest

The authors declare that they have no competing interests.

## Data Availability

Data will be made available on request.
