# Supplementary material for: NIR-II biomimetic nanoplatform optogenetic CD274 editing of HNSCC immunogenicity for enhanced photoimmunotherapy
Source: Mater Today Bio. 2026 Jan 13;37:102803. doi: 10.1016/j.mtbio.2026.102803 (PMC12857282; doi:10.1016/j.mtbio.2026.102803)
Supplement: Multimedia component 1 [file mmc1.docx]

**Supporting Information**

**NIR-II biomimetic nanoplatform optogenetic CD274 editing of HNSCC immunogenicity for enhanced photoimmunotherapy**

*Yang Chen^a^, Longcai Liu^a^, Xiaojuan Hu^a^, Yilin Huang^a^, Shijie Yao^a^, Lichen Ji^c^, Hai Zou*^b^, Xiaozhou Mou*^a^, Yu Cai*^a^*

^a^Centre for Rehabilitation Medicine, Rehabilitation & Sports Medicine Research Institute of Zhejiang Province, Department of Rehabilitation Medicine, Cancer Centre, Zhejiang Provincial People's Hospital (Affiliated People's Hospital), Hangzhou Medical College, Hangzhou, Zhejiang, China, 310014.

^b^Department of Emergency and Critical Care Medicine, Pudong New Area People's Hospital Affiliated to Shanghai Jiao Tong University School of Medicine.

^c^Department of Joint Surgery, Shanghai East Hospital, Tongji University School of Medicine, Shanghai, 200092, China.

E-mail addresses: zouhai@fudan.edu.cn, mouxz@zju.edu.cn, iamycai@163.com

**Experimental section**

**Materials and methods**

All chemicals were obtained from commercial suppliers and used as received without further purification. DSPE-PEG_2000_-COOH was purchased from Aladdin (Shanghai, China). 2-Morpholinoethanesulfonic Acid (MES), 1-(3-Dimethylaminopropyl)-3-ethylcarbodiimide hydrochloride (EDC) and N-Hydroxysuccinimide (NHS) were purchased from Sigma-Aldrich. Cell Counting Kit (CCK-8) was purchased from Yeasen Science & Technology Co. Calcein/PI Cell Viability/Cytotoxicity Assay Kit, Annexin V-FITC Apoptosis Detection Kit, and Hoechst 33342 (1000×) were purchased from Beyotime Biotechnology Co., Ltd. Annexin V-FITC/propidium iodide was purchased from SONY. αLDLR was purchased from proteintech (Wuhan, China). The primary antibodies for Hsp70, CD274, Cas9, CRT, HMGB1 and GAPDH and secondary antibodies were purchased from Cell Signalling Technology (USA). Lipofectamine 2000 transfection reagent were purchased from Thermo Fisher Scientific. T7 endonuclease I (T7EI), T4 ligase, and T4 phosphokinase were purchased from Beyotime Biotechnology Co., Ltd. The antibodies used to perform flow cytometry were the following: PE/Cyanine7 Anti-Mouse CD8a Antibody[53-6.7], PerCP/Cyanine5.5 Anti-Mouse CD45 Antibody[30-F11], PerCP/Cyanine5.5 Anti-Mouse F4/80 Antibody[CI:A3-1], APC Anti-Mouse CD80 Antibody[16-10A1], PE Anti-Mouse CD3 Antibody[17A2], FITC Anti-Mouse CD4 Antibody[GK1.5], PE Anti-Mouse CD86 Antibody[GL-1], FITC Anti-Mouse CD11c Antibody[N418], Purified Anti-Mouse CD16/32 Antibody[2.4G2], Cell Staining Buffer, 10×ACK Lysis Buffer, all monoclonal antibodies, purchased from Elabscience Biotechnology Co., Ltd. IFN-γ, TNF-α, IL-6 and IL-1β ELISA kits were both purchased from Invitrogen (USA).

The morphology of PC, RPC and ARPC were examined by transmission electron microscope (TEM, Hitachi H-7650). The size distribution (diameter, nm) and surface charge (zeta potential, mV) of the nanoparticles were measured by the Zetasizer Nano ZS particle analyzer (Malvern Instruments Limited). The UV–vis absorption spectrums of nanoparticles were recorded using a UV–vis spectrophotometer (Thermo Fisher). The PA intensity and images were collected from TOMOWAVE (USA). Red blood cell membrane protein of ARPC was characterized by the sodium dodecyl sulfate-polyacrylamide gel electrophoresis (SDS-PAGE) method. Confocal microscopy (LEICA Stellaris sted) was used for detecting the successful modification of αLDLR on ARISP. The heat generated by the laser was monitored by a thermal imager camera (FLUKE, TI400).

**Construction of Hsp-Cas9**

we designed primers to obtain HSP70 promoter fragment from the upstream region of the mouse HSP70 protein gene by PCR amplification. The guide RNA, prime and Plasmid pX458 were purchased from GenScript Biotech Corporation (Nanjing, China). And these sequences can be viewed in Supporting Information.

**Preparation of RBCm**

In brief, whole blood was collected from Sprague-Dawley (SD) rats via the abdominal aorta using a syringe, then centrifuged at 2000 rpm for 10 minutes at 4 °C. The serum and buffy coat were carefully removed, and the erythrocytes were collected and washed three times with cold PBS (1×). The packed erythrocytes were then re-collected by centrifugation. The washed erythrocytes were suspended in a hypotonic solution (0.25 × PBS) and incubated on ice for 20 minutes, followed by centrifugation at 12,000 rpm for 5 minutes, repeated three times to remove hemoglobin.

**Synthesis of DSPE-PEG_2000_-αLDLR**

DSPE-PEG_2000_-COOH (600 µL, 10 mg mL^-1^, dissolved in MES) was activated through reaction with NHS (67.5 µL, 0.25 M) and EDC (67.5 µL, 0.25 M) for 30 minutes at room temperature, followed by neutralization to pH 7.5 with sodium hydroxide. The αLDLR antibody was then incubated with the activated DSPE-PEG_2000_-COOH at 4 °C overnight. The resulting conjugate was dialyzed using 3500 MWCO dialysis tubing for 24 hours in PBS to remove unincorporated DSPE-PEG_2000_-COOH. This was followed by further dialysis with 300 kDa dialysis tubing for 4 hours in PBS to eliminate unincorporated antibodies. An additional 4 hours of dialysis was performed to remove and refresh the PBS. The purified DSPE-PEG_2000_- αLDLR was then stored at 4 °C for future use.

**Preparation of PC**

PLGA (100 mg) was dissolved in ethyl acetate (5 mL), and PT (10 mg) was dissolved in THF and Hsp-Cas9 was dissolved into DEPC water (1 mL). To form the PT-Hsp-Cas9 (PC) core, the other solutions were added dropwise into the PLGA solution and ultrasound at 20% W for 5 min to get the initial emulsion. Then, the initial emulsion was mixed with 2% PVA solution and ultrasound at 20% W for 10 min to get multiple emulsions. The multiple emulsion was added with 0.5% PVA and ultrasound at 20% W for 10 min to stabilize the emulsion.

**Preparation of ARPC**

DSPE-PEG_2000_-αLDLR was added into RBCm vesicles and reacted overnight at 4 °C to modify the membrane. The αLDLR modified erythrocyte membrane vesicles were collected by centrifugation at 3500 rpm for 10 min at 4 °C. The prepared PC was dissolved in PBS solution containing DSPE-PEG_2000_-αLDLR, and then the mixture was sonicated in an ice bath for 20 min, and finally incubated overnight at 4 °C on a shaker. The next day, the excess erythrocyte membrane was removed by centrifugation, getting ARPC. The same procedures with PC core prepared the (RBCm-PT-Hsp-Cas9) RPC, but RBCm without any modification to make sure the only difference between the ARPC and the RPC was the LDLR targeting. Subsequently, the size distribution and ζ potential of the nanocomplex were detected by dynamic light scattering. The morphology and mapping of the sample was further characterized by transmission electron microscopy. Red blood cell membrane protein of ARPC was characterized by the sodium dodecyl sulfate-polyacrylamide gel electrophoresis (SDS-PAGE) method.

**Photothermal performance of ARPC in solution**

Prepare ARPC aqueous solutions at different concentrations (0, 37.5, 75, 150, 300 μg/mL), a 1060 nm near-infrared laser with power density gradients of 1, 0.75, 0.5 W/cm^2^, quartz cuvettes, and an infrared thermal imager, among other materials and equipment. Then, conduct photothermal effect tests for ARPC at different concentrations, that is, prepare solutions at each concentration, place them in quartz dishes, irradiate with a 1060 nm laser at a power density of 0.75 W/cm^2^ for 10 minutes, and record the temperature changes in real time using an infrared thermal imager. Subsequently, carry out photothermal effect tests at different laser power densities, prepare 1 mL of 300 μg/mL ARPC aqueous solution in a quartz cuvette, irradiate with lasers at different power densities for 10 minutes, and record the temperature. And perform photothermal stability tests by subjecting the 300 μg/mL ARPC aqueous solution to three cycles with a laser at a power density of 1 W/cm^2^, recording the temperature every 30 seconds. Finally, calculate the photothermal conversion efficiency to evaluate the photothermal conversion capability, concentration, power dependence, and stability of ARPC.

$$\eta=\frac{\mathrm{hA}\left( {\Delta T}_{max, ARPC}-{\Delta T}_{max, H_{2}O} \right)}{I\left( 1-{10}^{-A\lambda} \right)}$$

Where *h* is the heat transfer coefficient, *A* is the surface area of the container, *∆T_max,ARPC_*, and *∆T_max_,_H2O_* is the temperature change of ARPC dispersion and water at the maximum steady-state temperature, *I* is the laser power, *A_λ_* is the absorbance of ARPC at the wavelength of 1060 nm in aqueous solution. In this study, the photothermal conversion efficiency of ARPC was calculated of 39.32%.

**Targeted Cellular Uptake and Distribution of ARPC**

SCC7 cells were co-incubated with PC, RPC and ARPC in cell plates for different times. To facilitate tracking the nanoparticle, the plasmid Hsp-Cas9 was labelled with YOYO-1 Iodide and RBCm was labelled with DID. And then, the cells were collected and analyzed by CLSM and FACS. In order to observe the distribution of ARPC, the SCC7 cells were co-cultured with YOYO-labelled ARPC for different times. Then, the cells were stained by LysoTracker Red and Houchst according the standard protocol provided by the manufacturer. Subsequently, the samples were visualized under CLSM scanning confocal.

**ARPC Transfection In Vitro**

SCC7 cells in a 12-well plate were incubated with PC, RPC and ARPC for 24 h. The treated cells were incubated for another 24 h after stimulation with 1060 nm laser irradiation. Finally, the expression of the GFP protein in SCC7 cells was observed with CLSM. The SCC7 cells were collected by trypsin treatment and then the transfection efficiency of SCC7 cells was detected by FACS.

**CD274 Disruption In Vitro**

First, the Hsp-Cas9 plasmid transfected by Lipofectamine 2000 Transfection Reagent was used to analysis of heating time conditions. Additionally, the SCC7 cells in a 12-well plate were treated with PBS, PC, RPC, ARPC, PBS+Laser, PC+Laser, RPC+Laser, and ARPC+Laser for 48 h. The cells were incubated with IFN-γ to mimic the condition that in tumors, interferon-γ (IFN-γ) is produced by tumor-infiltrating cytotoxic T lymphocytes (CTLs) and induces the expression of CD274 on tumor cells. After another 24 h of culture, the cells were collected into 1.5 mL EP tubes and labelled with APC-CD274 antibody for flow cytometry analysis. The procedure for detecting CD274, Hsp70 and Cas9 protein expression level by Western blot and CLSM.

**Immunogenic Cell Death of SCC7 Cells In Vitro**

Similar to the aforementioned experimental procedure, SCC7 cells were incubated with PBS, PT, PC, RPC, ARPC, PBS+Laser, PT+Laser, PC+Laser, RPC+Laser, and ARPC+Laser.

After 24 h of light irradiation, the culture supernatant was collected into a 1.5 mL centrifuge tube, while the cells in well plate were digested and collected into 1.5 mL centrifuge tubes. Quantification of the respective indicators was performed using a ELISA for IL-6, IL-1β, TNF-α, and IFN-γ. The amount of ATP released in culture supernatant of the medium was detected using an ATP Determination Kit (Beyotime Biotechnology) following the manufacturer’s protocol. Then the cells were labelled with AlexaFluor 488 conjugated anti-calreticulin and anti-HMGB1 antibody, and the level of CRT and HMGB1 was detected by CLSM and WB. The amount of ATP released in culture supernatant of the medium was detected using an ATP Determination Kit (Beyotime Biotechnology) following the manufacturer’s protocol.

**T Cell Proliferation after ICD In Vitro**

In line with the previously described experimental procedures, SCC7 cells were treated with PBS, PT, PC, RPC, ARPC, PBS+Laser, PT+Laser, PC+Laser, RPC+Laser, and ARPC+Laser. The pretreated SCC7 cells were then co-cultured with T cells (anti-CD8+ labelled with PE/Cyanine7) for 48 hours. Subsequently, the cells were collected into 1.5 mL EP tubes for flow cytometry analysis. Additionally, the levels of TNF-α, IL-6, and IL-1β in culture supernatant were measured using the Mouse TNF-α, IL-6, and IL-1β ELISA Kit.

**M1 Cells after ICD In Vitro**

In accordance with the previously described experimental procedures, SCC7 cells were treated with various conditions including PBS, PT, PC, RPC, ARPC, PBS+Laser, PT+Laser, PC+Laser, RPC+Laser, and ARPC+Laser. The pretreated SCC7 cells were then co-cultured with RAW cells (anti-CD80 labelled with APC and anti-CD86 labelled with PE) for 48 hours. Following co-culturing, the cells were harvested into 1.5 mL EP tubes for flow cytometry analysis. Additionally, the levels of TNF-α, IL-6, and IL-1β in culture supernatant were assessed using the Mouse TNF-α, IL-6, and IL-1β ELISA Kit.

**DC Maturation In Vitro**

Following the experimental protocol, SCC7 cells were treated with various conditions including PBS, PT, PC, RPC, ARPC, PBS+Laser, PT+Laser, PC+Laser, RPC+Laser, and ARPC+Laser. The pretreated SCC7 cells were then combined with RAW cells (anti-CD45 labelled with PerCP/Cyanine5.5, anti-CD11c labelled with FITC, anti-CD80 labelled with APC and anti-CD86 labelled with PE) and co-cultured for 48 hours. Subsequently, the cells were collected into 1.5 mL EP tubes for flow cytometry analysis. Additionally, the levels of TNF-α, IL-6, and IL-1β in culture supernatant were measured using the Mouse TNF-α, IL-6, and IL-1β ELISA Kit.

**SCC7 Primary Tumor Model**

Female Balb/c mice, aged 6 weeks, were procured from GemPharmatech Co., Ltd. A suspension of 5×10^7^ SCC7 cells was injected into the cricothyroid region of the throat. After 10 days, the mice were randomly assigned to one of five groups (n = 8). Various formulations (PBS, ARP, ARP+L, ARPC, ARPC+L) were administered via peritumoral injection every other day. The tumors were subsequently irradiated 24 hours after injection with a 1060 nm laser (0.75 W/cm^2^) for 9 minutes. Tumor volume and body weight of the mice were recorded throughout the treatment period. Additionally, the experimental protocol for assessing the antitumor efficacy of ARPC was conducted as described previously.

**CD274 Disruption In Vivo**

In brief, tumors from mice in each experimental group were harvested following the completion of the various treatments. Consistent with the in vitro detection methods, the expression levels of CD274 and Hsp70 proteins in tumors were assessed using Western blot, immunofluorescence, and flow cytometry (FACS).

**Flow Cytometry**

Following treatment, the tumor was excised and immediately sectioned, with a suitable quantity placed into a 5 mL EP tube. The tumor was fragmented with scissors and subsequently treated with 3 mL of digestion solution (0.1 mg/mL DNase, 0.1 mg/mL hyaluronidase, and 1.0 mg/mL collagenase) at 37 °C for 10 minutes. The digested tumor tissue was then filtered through a 200-mesh steel sieve to obtain a single-cell suspension. The resulting cell pellet was collected and purified using 6 mL of 40% Percoll, followed by the addition of 2 mL of red blood cell lysate to eliminate red blood cells. Finally, the isolated cell pellet was stained with antibodies and subjected to flow cytometry analysis.

**Ethical Statement**

All animal experiment procedures were reviewed and approved by Laboratory animal management and ethics committee of Zhejiang Provincial People’s Hospital (ZPPH) and the approval No. of the animal ethical is “20231205205155895636”.


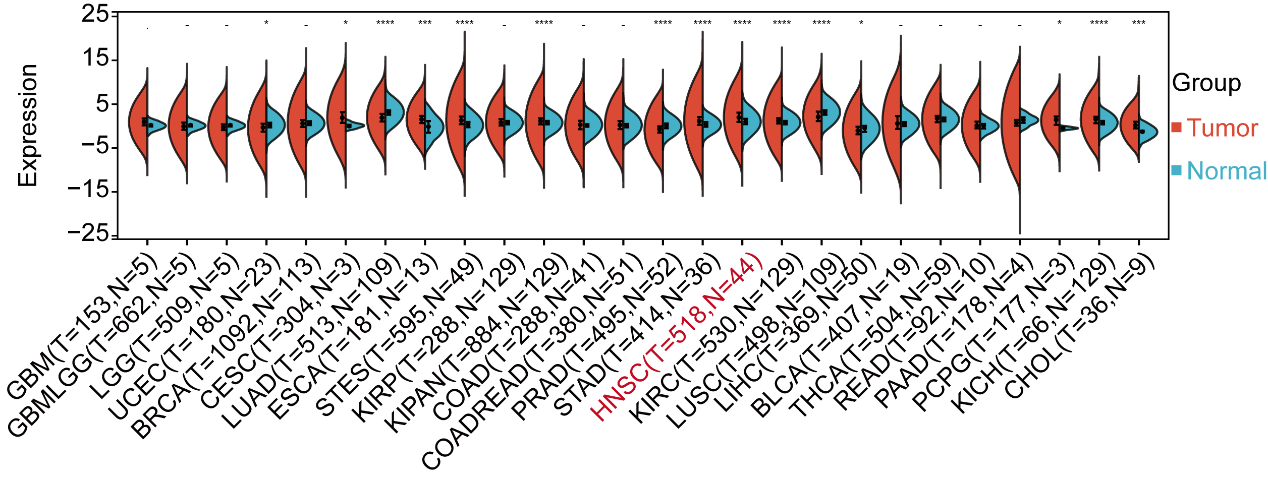
**Figure S1.** Violin plot showing expression of CD274 in each cancer type.

**Table S1.** Primer sequences for sgRNA synthesis used in this study.

| Primer names | Sequences (5’-3’) |
| --- | --- |
| CD274 sg1 | GCTGGACCTGCTTGCGTTAG |
| PD-L1Sg1 PROMOTER eSpCas9-2A- GFP(PX458) |  |
| CD274 sg2 | GCTTGCGTTAGTGGTGTACT |
| PD-L1Sg2 PROMOTER eSpCas9-2A- GFP(PX458) |  |
| CD274 sg3 | GAACTAATATGTCAGGCCGA |
| PD-L1Sg3 PROMOTER eSpCas9-2A- GFP(PX458) |  |

**Table S2.** The sequencing of Hsp70 promoter in the plasmid.

| Hsp70 Promoter |
| --- |
| CACTCTGGCCTCTGATTGGTCCAAGGAAGGCTGGGGGGCAGGACGGGAGGCGAAAACCCTGGAATATTCCCGACCTGGCAGCCTCATCGAGCTCGGTGATTGGCTCAGAAGGGAAAAGGCGGGTCTCCGTGACGACTTATAAAAGCCCAGGGGCAAGCGGTCCGGATAACGGCTAGCCTGAGGAGCTGCTGCGACAGTCCACTACCTTTTTCGAGAGTGACTCCCGTTGTCCCAAGGCTTCCCAGAGCGAACCTGTGCGGCTGCAGGCACCGGCGCGTCGAGTTTCCGGCGTCCGGAAGGACCGAGCTCTTCTCGCGGATCCAGTGTTCCGTTTCCAGA |


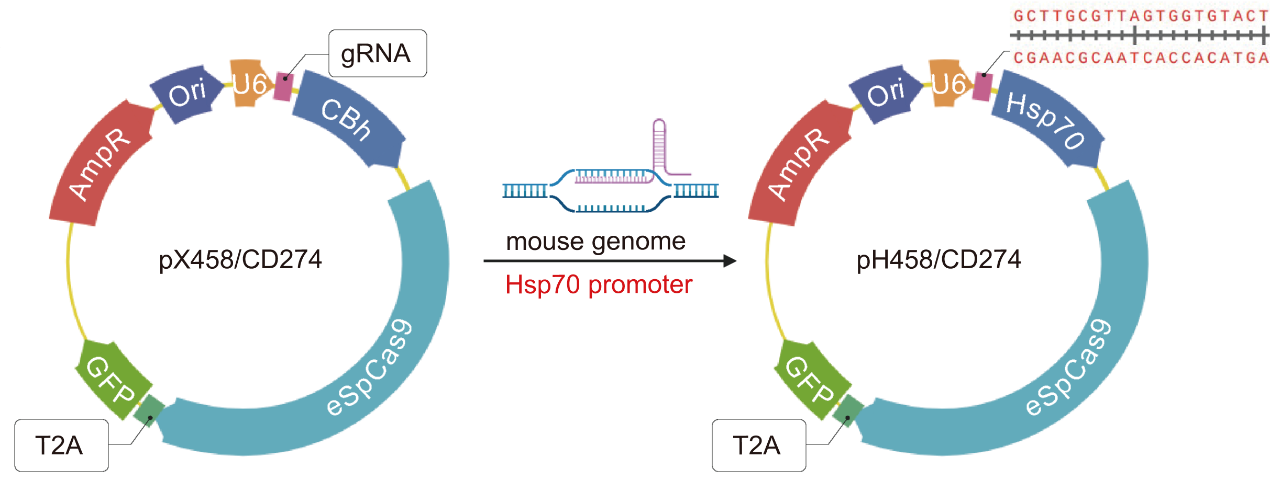


**Figure S2.** Schematic representation of the Hsp70 promoter-driven Hsp-Cas9 pH458/sgCD274.


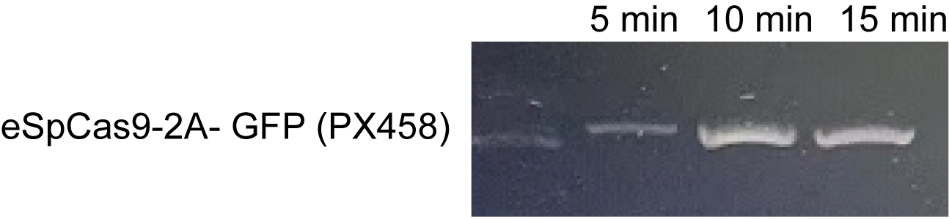


**Figure S3.** Regarding the analysis of the results of eSpCas9-2A-GFP (PX458) transfection using T7 Endonuclease I.


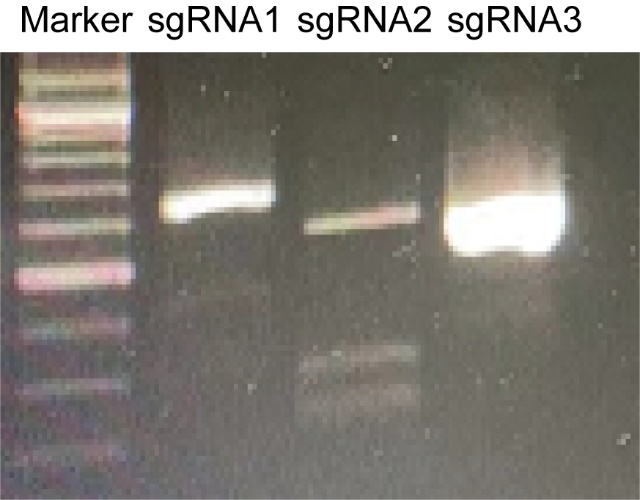


**Figure S4.** Regarding the analysis of the results of sgRNA1, sgRNA2 and sgRNA3 transfection using T7 Endonuclease I.


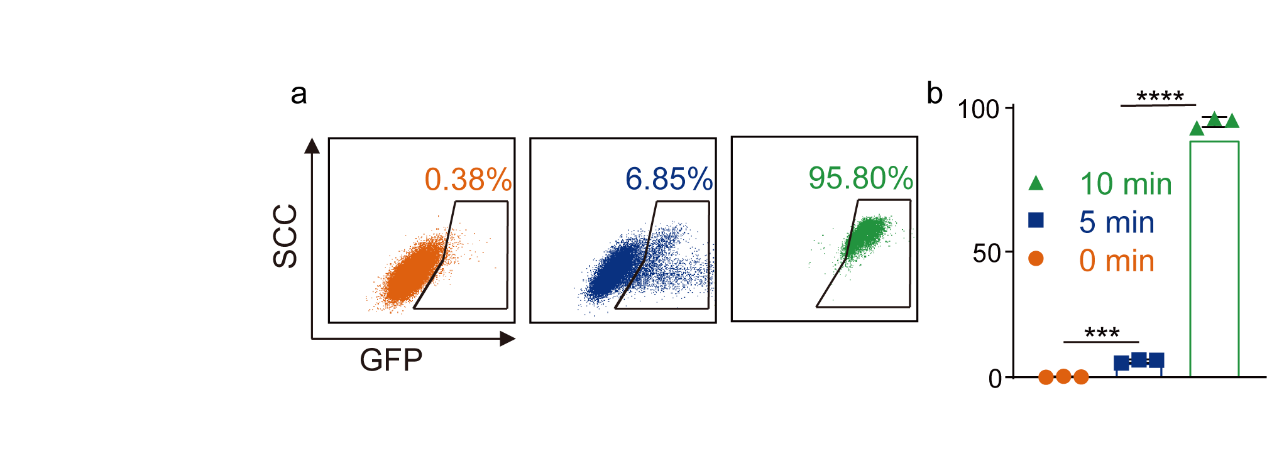


**Figure S5.** Flow cytometry analysis of GFP protein expression in SCC7 cells following transfection with Lipofectamine 2000.


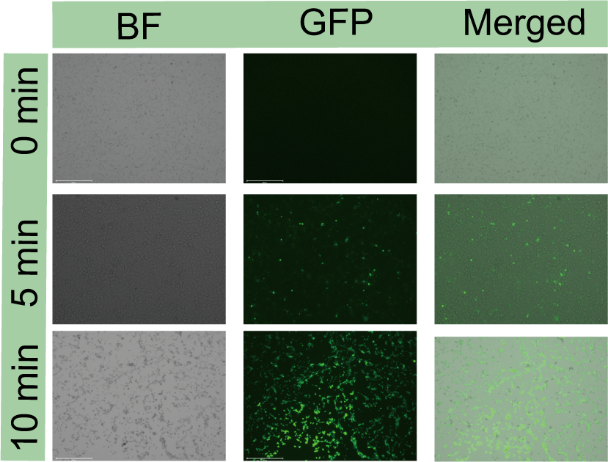


**Figure S6.** Each individual fluorescence channel of GFP.


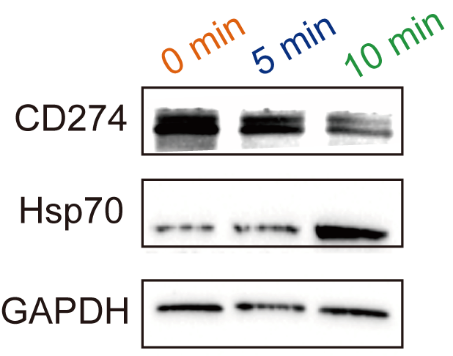


**Figure S7.** The expression levels of Hsp70 and CD274 in SCC7 cells after treatments, as assessed by Western blot.


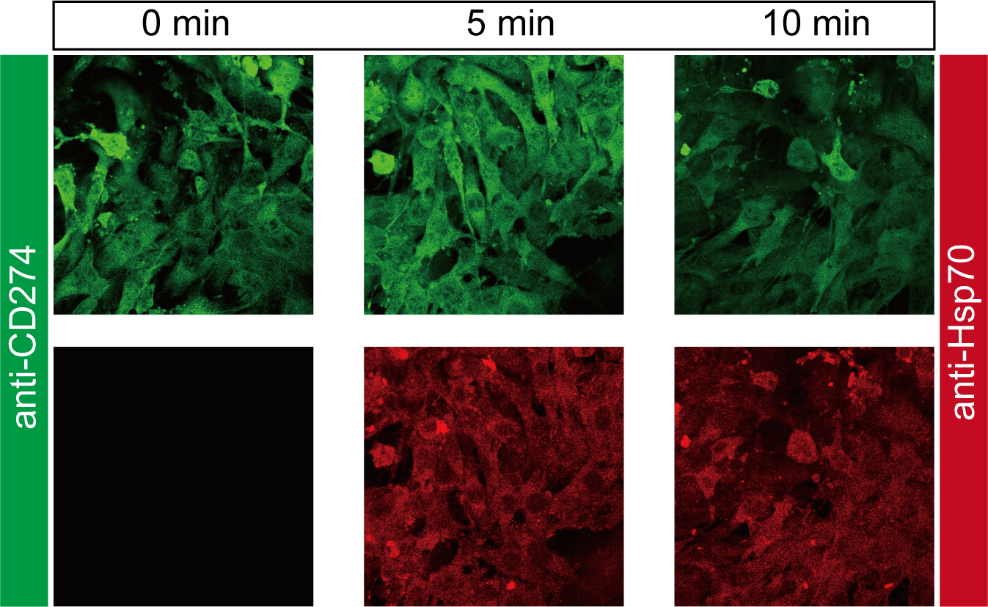


**Figure S8.** Each individual fluorescence channel of CD274 and Hsp70.


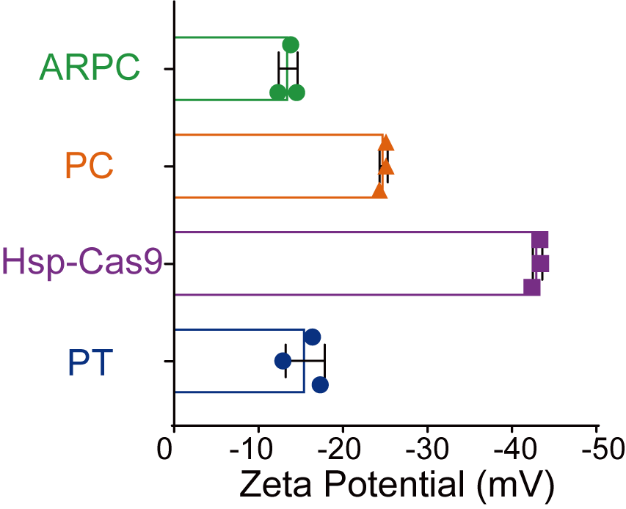


**Figure S9**. Zeta potential analysis of PT, Hsp-Cas9, PC, ARPC. Mean ± S.D., n = 3.


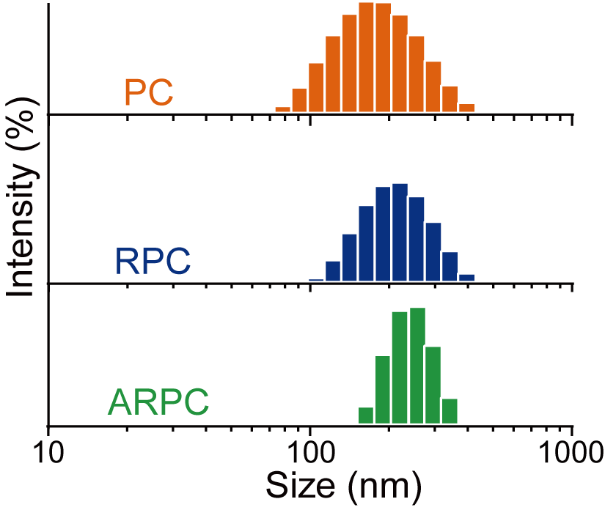


**Figure S10.** Hydrodynamic diameters of PC, RPC, ARPC.


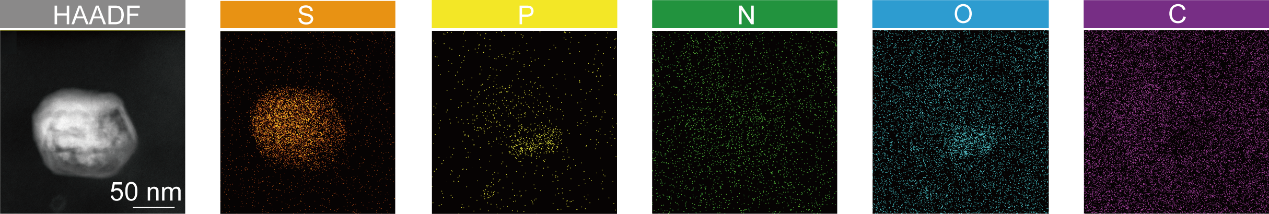


**Figure S11.** Elemental mapping of PC.


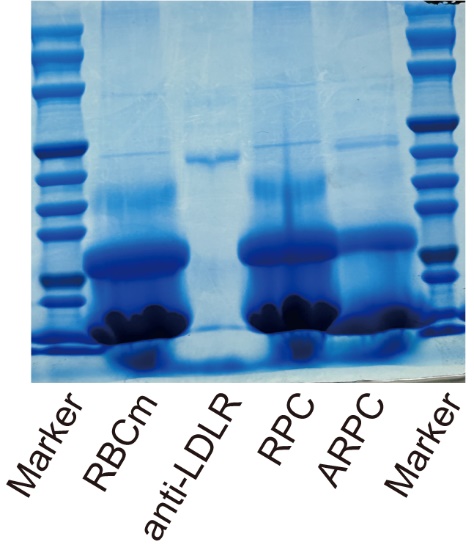


**Figure S12**. SDS-PAGE protein analysis.


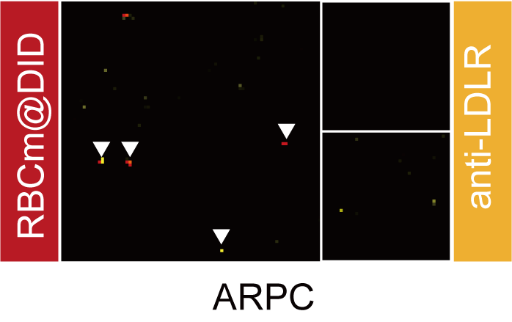


**Figure S13.** Colocalization analysis by CLSM observation over red fluorescence of DID in DID labelled RBCm, and yellow fluorescence of IgG H&L (Alexa Fluor™ Plus 555) antibody in antibody connected αLDLR.


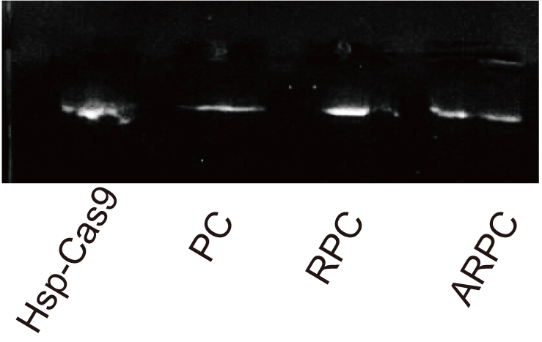


**Figure S14.** Agarose gel electrophoresis of Hsp-Cas9, PC, RPC and ARPC.


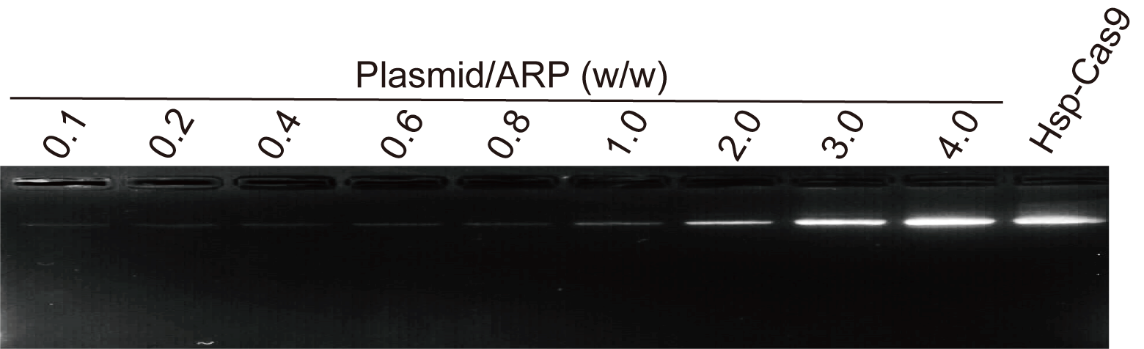


**Figure S15.** Agarose gel electrophoresis of ARPC nanocomplexes at various Hsp-Cas9/ARP ratios.


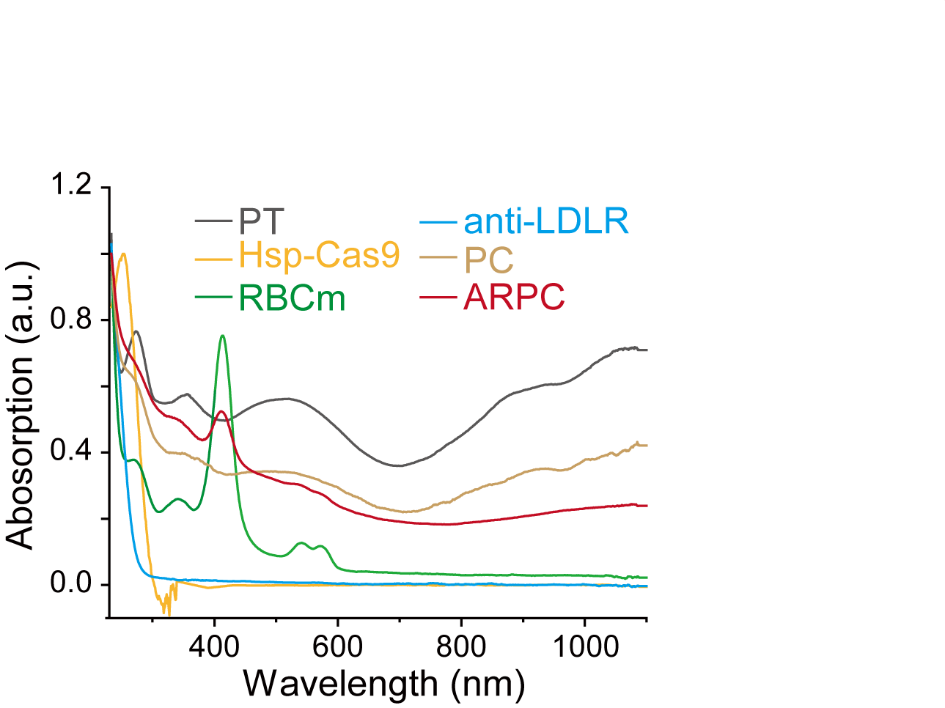


**Figure S16.** UV–vis spectra of PT, Hsp-Cas9, RBCm, αLDLR, PC and ARPC.


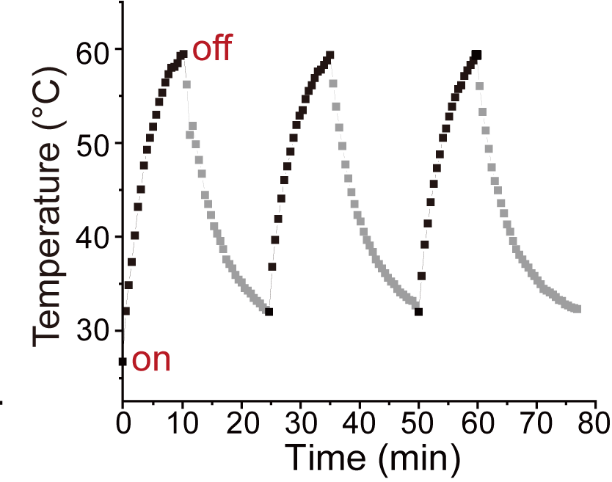


**Figure S17.** After three laser on/off cycle of laser irradiation (300 μg/mL, 1060 nm, 1 W/cm^2^), the change of temperature.


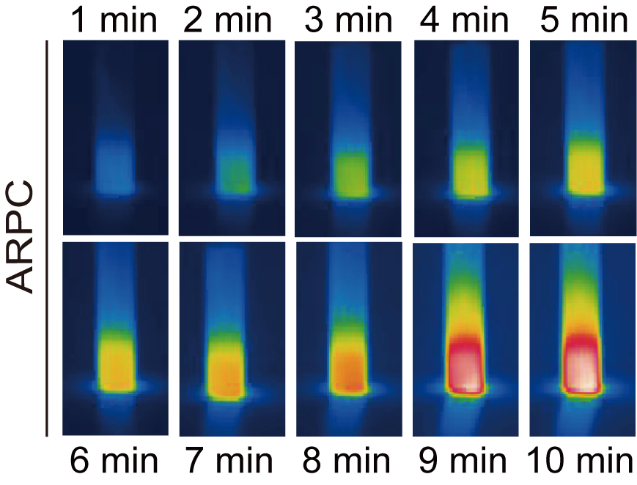


**Figure S18.** IR images of ARPC under laser irradiation for 10 min.


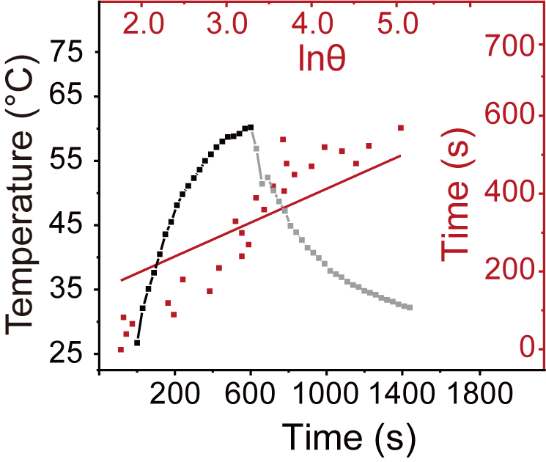


**Figure S19.** The calculated photothermal-conversion efficiency upon 1060 nm irradiation.


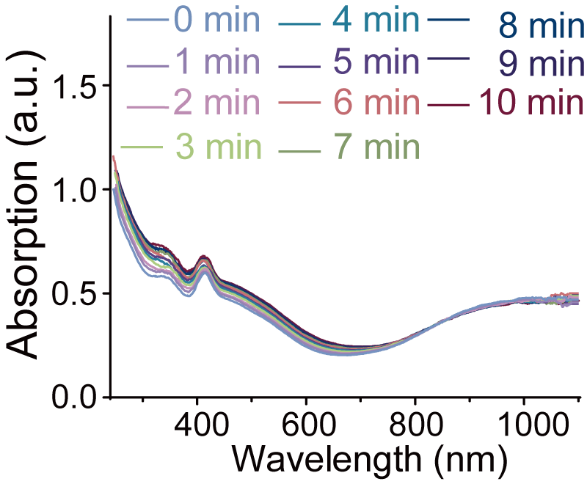


**Figure S20.** The UV absorbance variation of ARPC under continuous laser irradiation for 10 minutes.


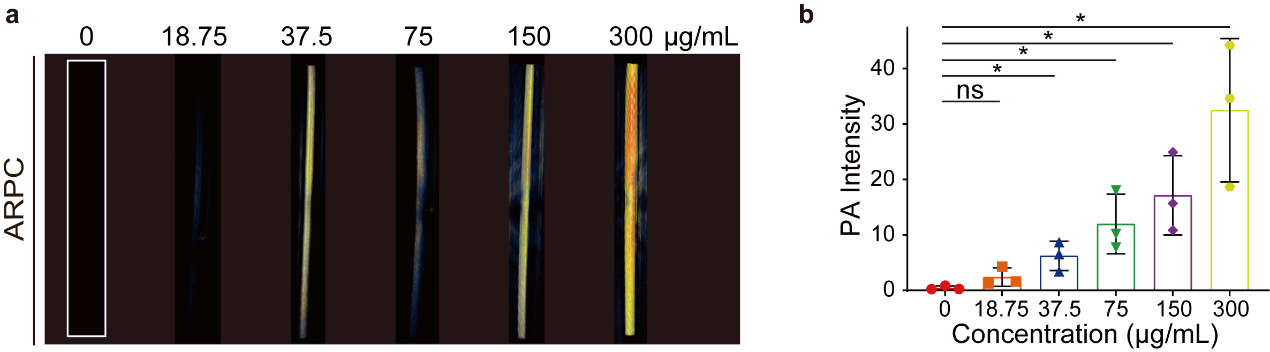


**Figure S21.** a) PAI of ARPC with series of concentrations ranged from 0 to 300 μg/mL. b) PAI intensity of ARPC with series of concentrations ranged from 0 to 300 μg/mL. Mean ± S.D., n = 3. *P < 0.05; **P < 0.01; ***P < 0.001; ****P < 0.0001.


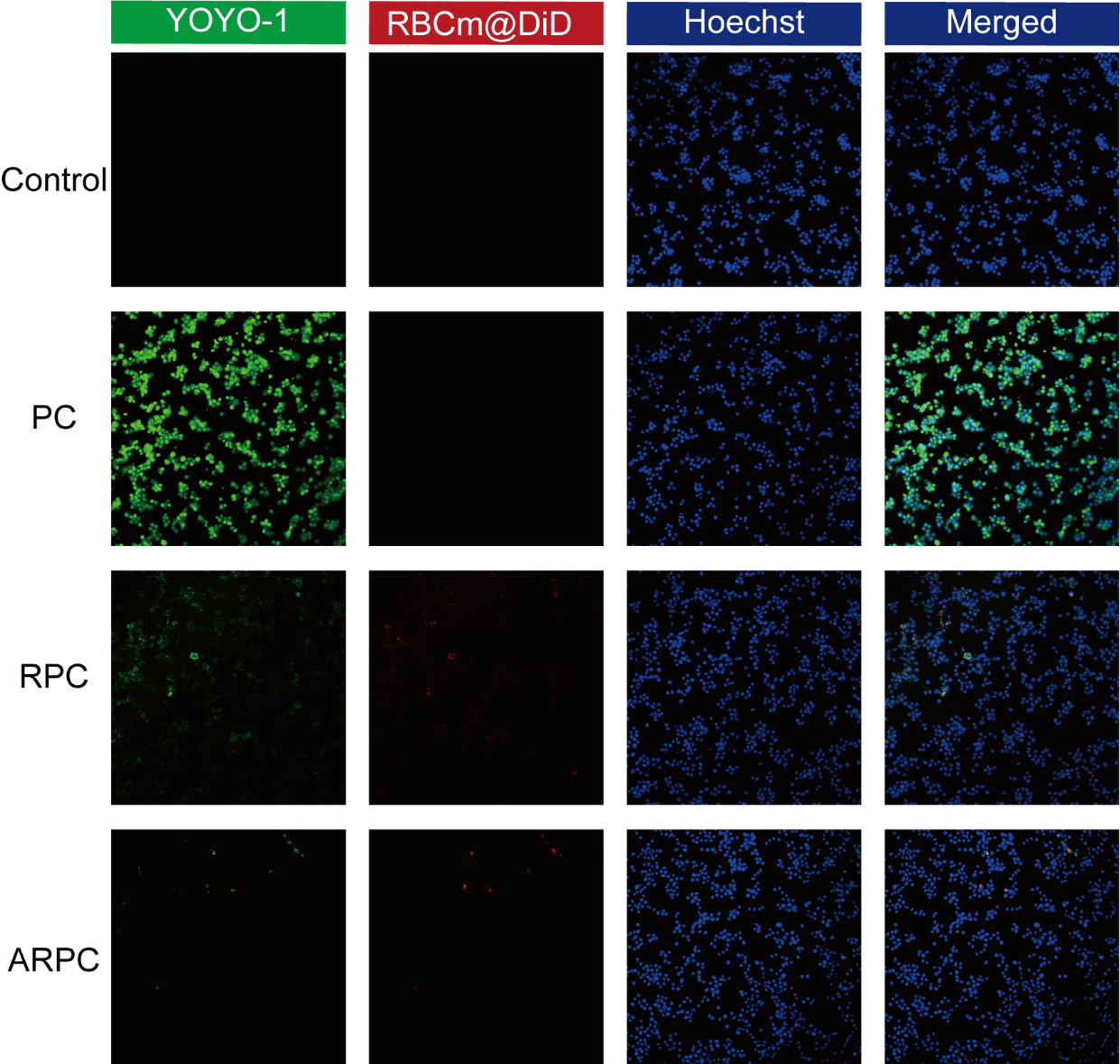


**Figure S22.** Each individual fluorescence channel of CLSM images in RAW264.7 cells after incubation with PC, RPC and ARPC.


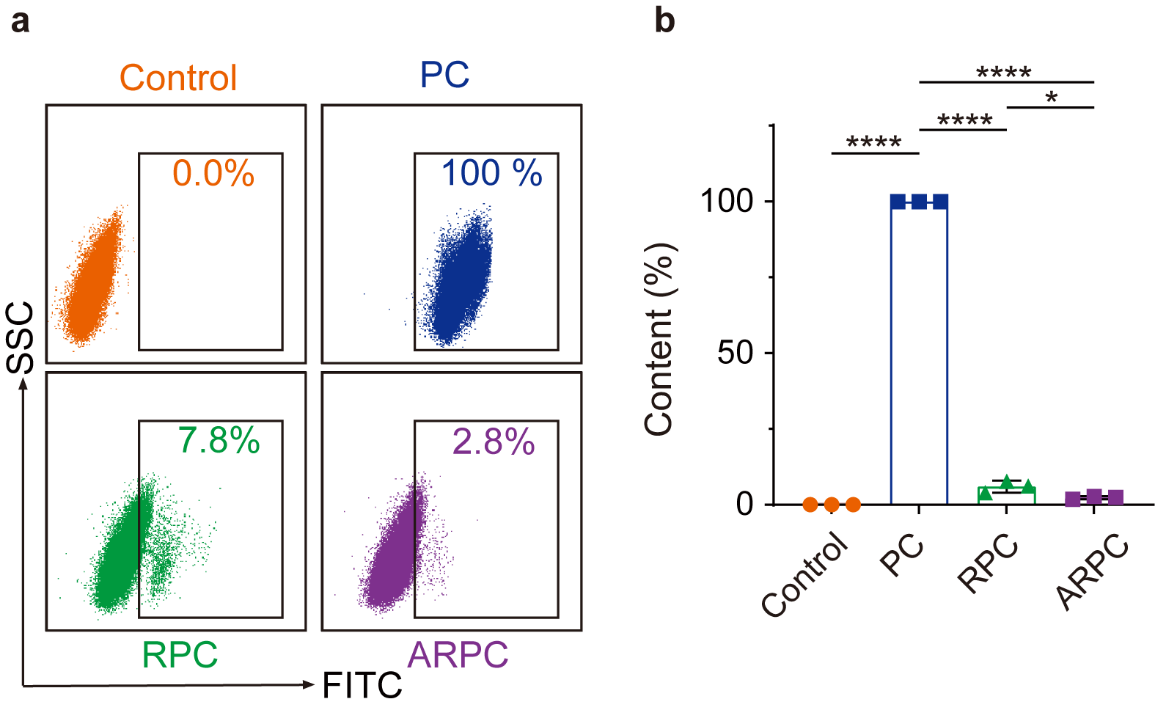


**Figure S23.** a) Cell flow cytometer in RAW264.7 cells after incubation with PC, RPC and ARPC. b) The statistical chart of the flow cytometry results. Mean ± S.D., n = 3. *P < 0.05; **P < 0.01; ***P < 0.001; ****P < 0.0001.


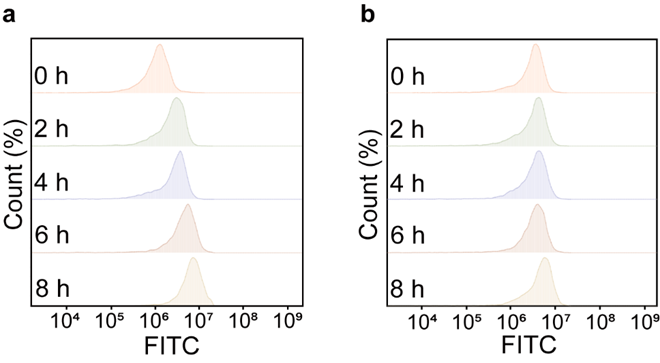


**Figure S24.** a) Flow cytometric analysis of ARPC (ARPC@YOYO-1) in RAW264.7 cells with different times. b) Flow cytometry quantitative analysis of DC2.4 cells treated with ARPC (ARPC@YOYO-1) for different incubation times.


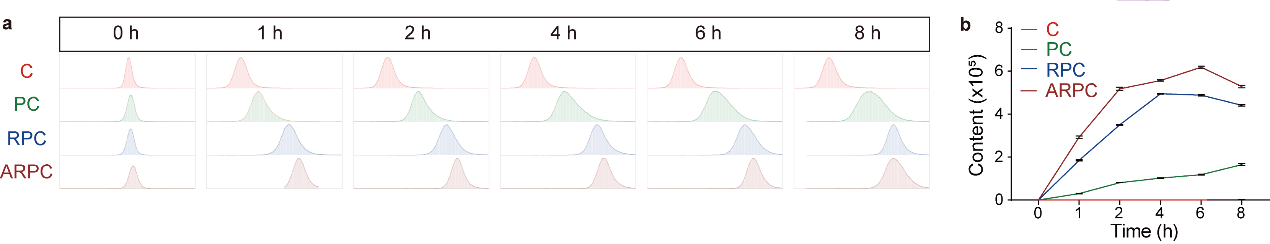


**Figure S25.** a) Flow cytometric analysis of C (CRISPR/Cas9@YOYO-1), PC (PC@YOYO-1), RPC (RPC@YOYO-1), ARPC (ARPC@YOYO-1) in SCC7 cells with different times. b) Flow cytometry quantitative analysis of SCC7 cells treated with C (CRISPR/Cas9@YOYO-1), PC (PC@YOYO-1), RPC (RPC@YOYO-1), ARPC (ARPC@YOYO-1) for different incubation times.


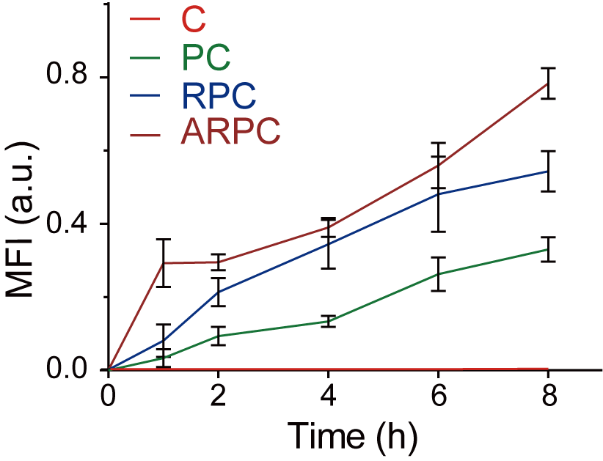


**Figure S26.** Quantitative analysis of fluorescent intensity in SCC7 cells incubated with C (CRISPR/Cas9@YOYO-1), PC (PC@YOYO-1), RPC (RPC@YOYO-1), ARPC (ARPC@YOYO-1).


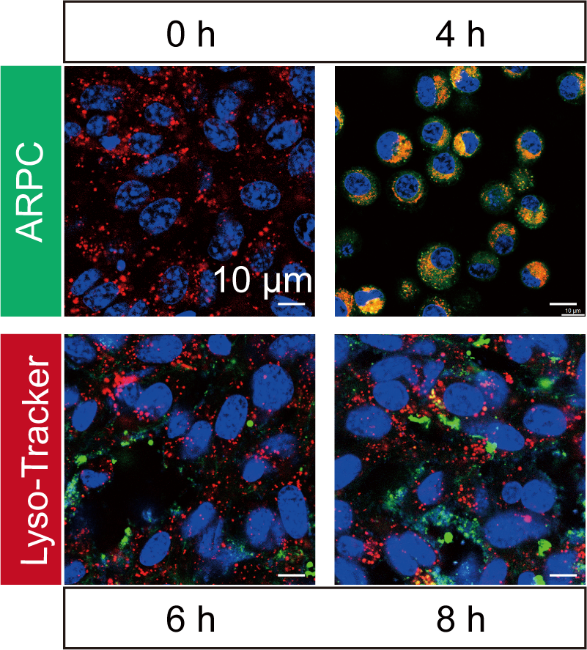


**Figure S27.** Intracellular distribution of ARPC by CLSM. Nucleus and endosome were stained with Houchst (blue) and Lyso-Tracker (red), and CRISPR/Cas9 was labelled with YOYO-1 iodide (green). Scale bar = 10 μm.


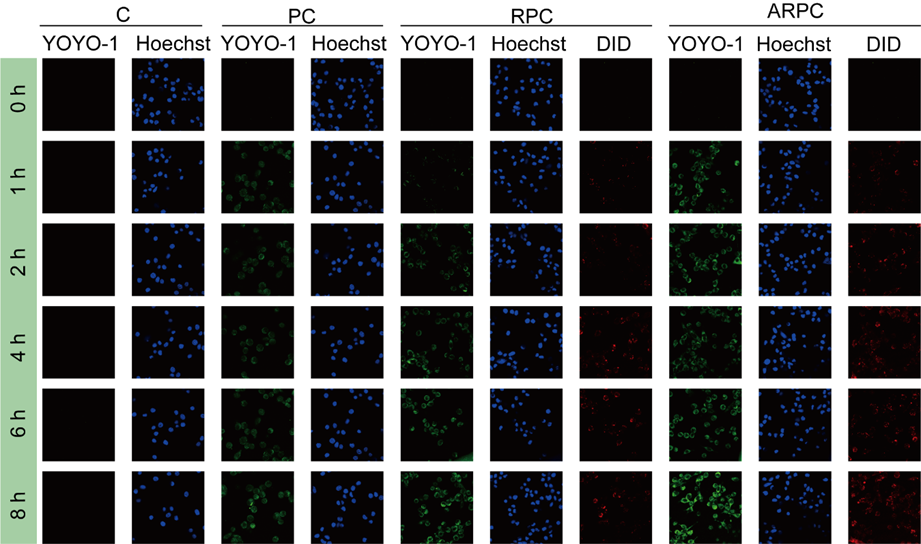


**Figure S28.** Each individual fluorescence channel of uptaking in SCC7 cells.


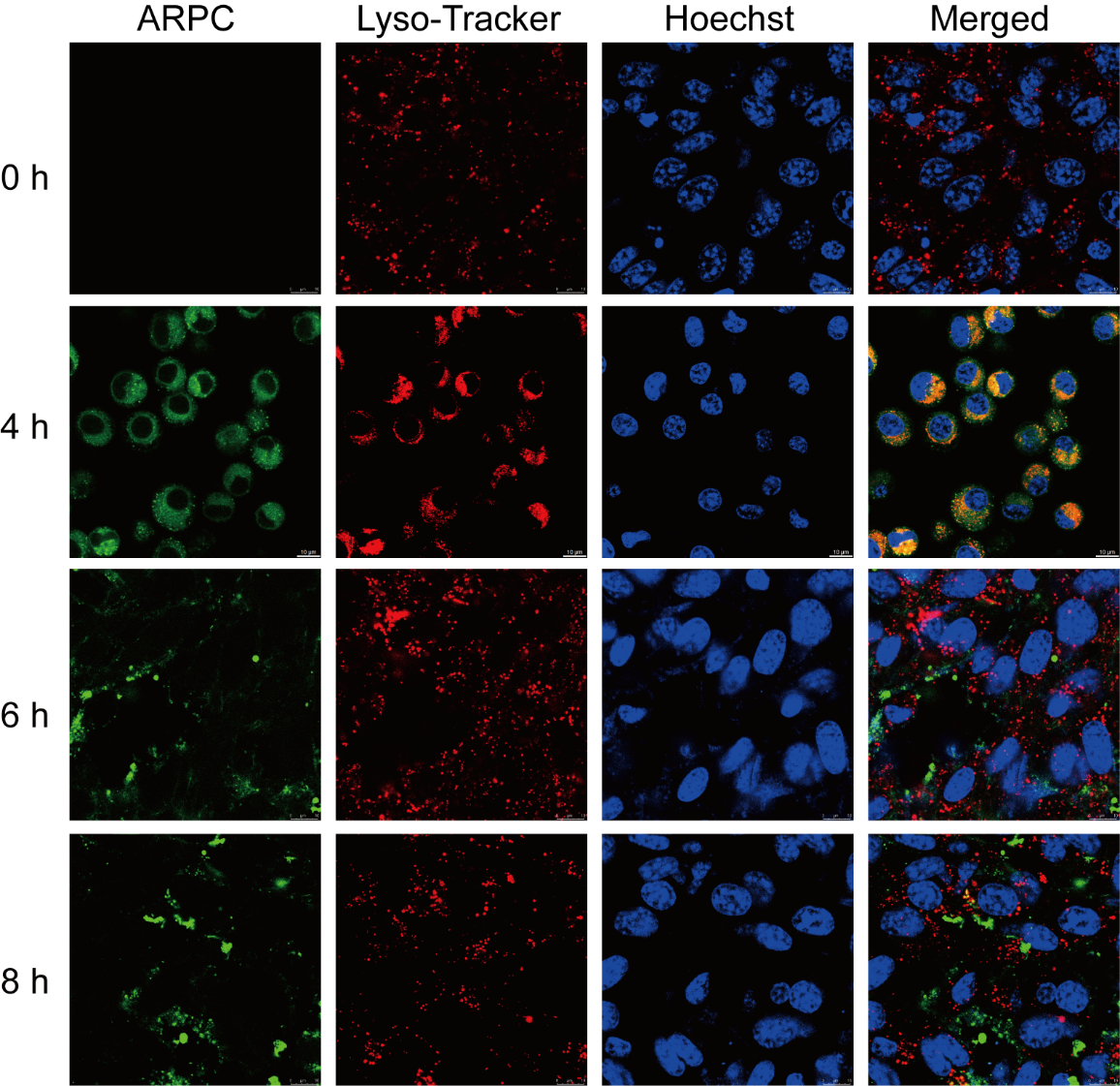


**Figure S29.** Each individual fluorescence channel of intracellular distribution in SCC7 cells.


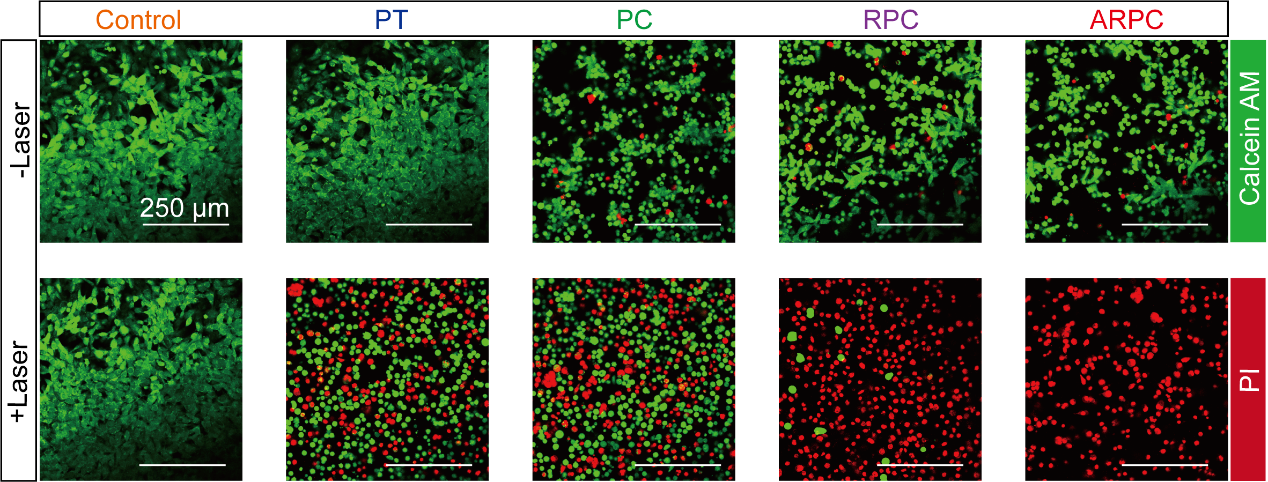


**Figure S30.** After exposing different groups to laser irradiation (1060 nm, 0.75 W/cm²), fluorescence images of live cells (green) and dead cells (red) were captured using confocal laser scanning microscopy (CLSM) (Scale bar = 250 μm).


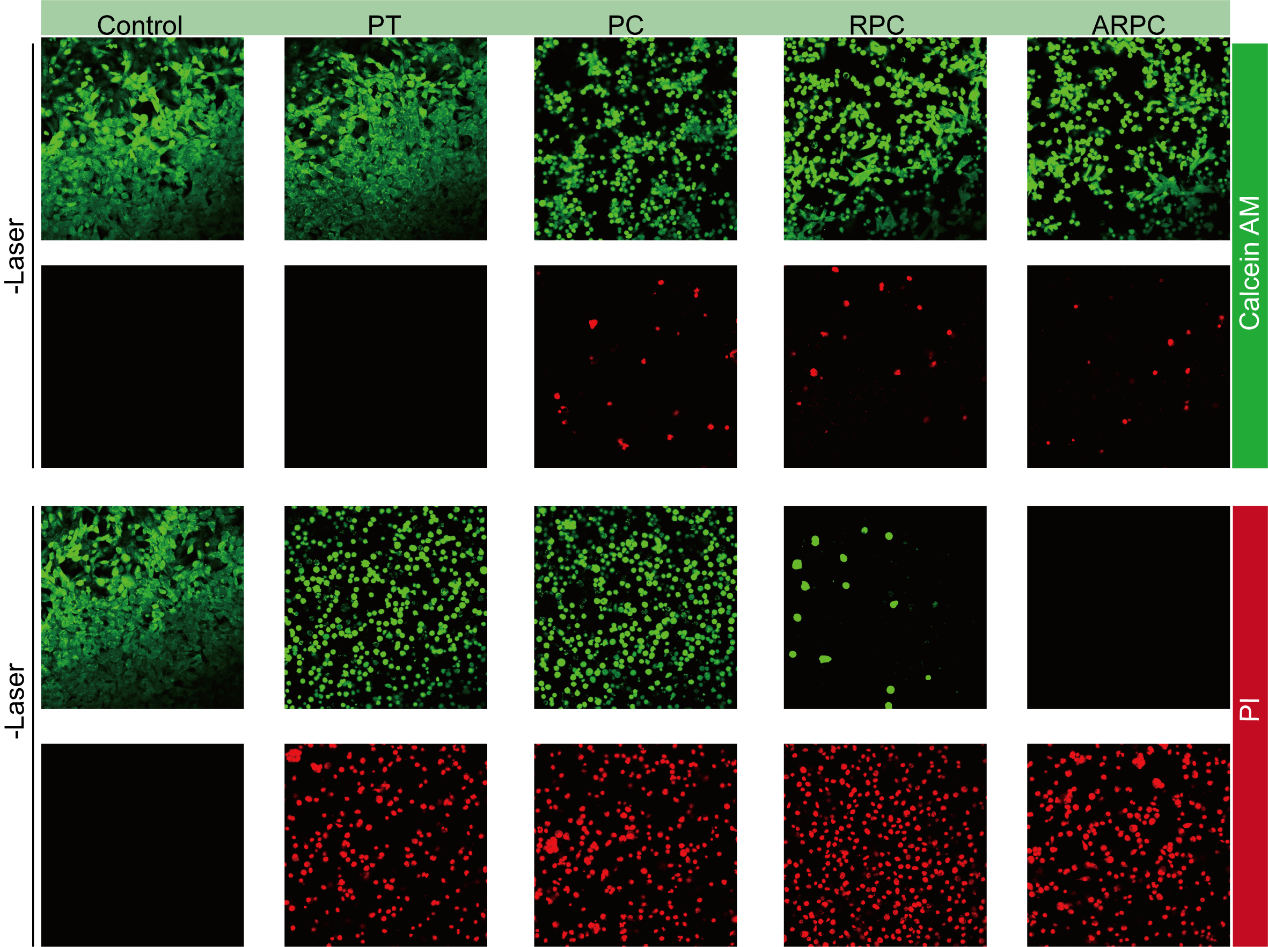


**Figure S31.** Each individual fluorescence channel of live cells (green) and dead cells (red) in SCC7 cells.


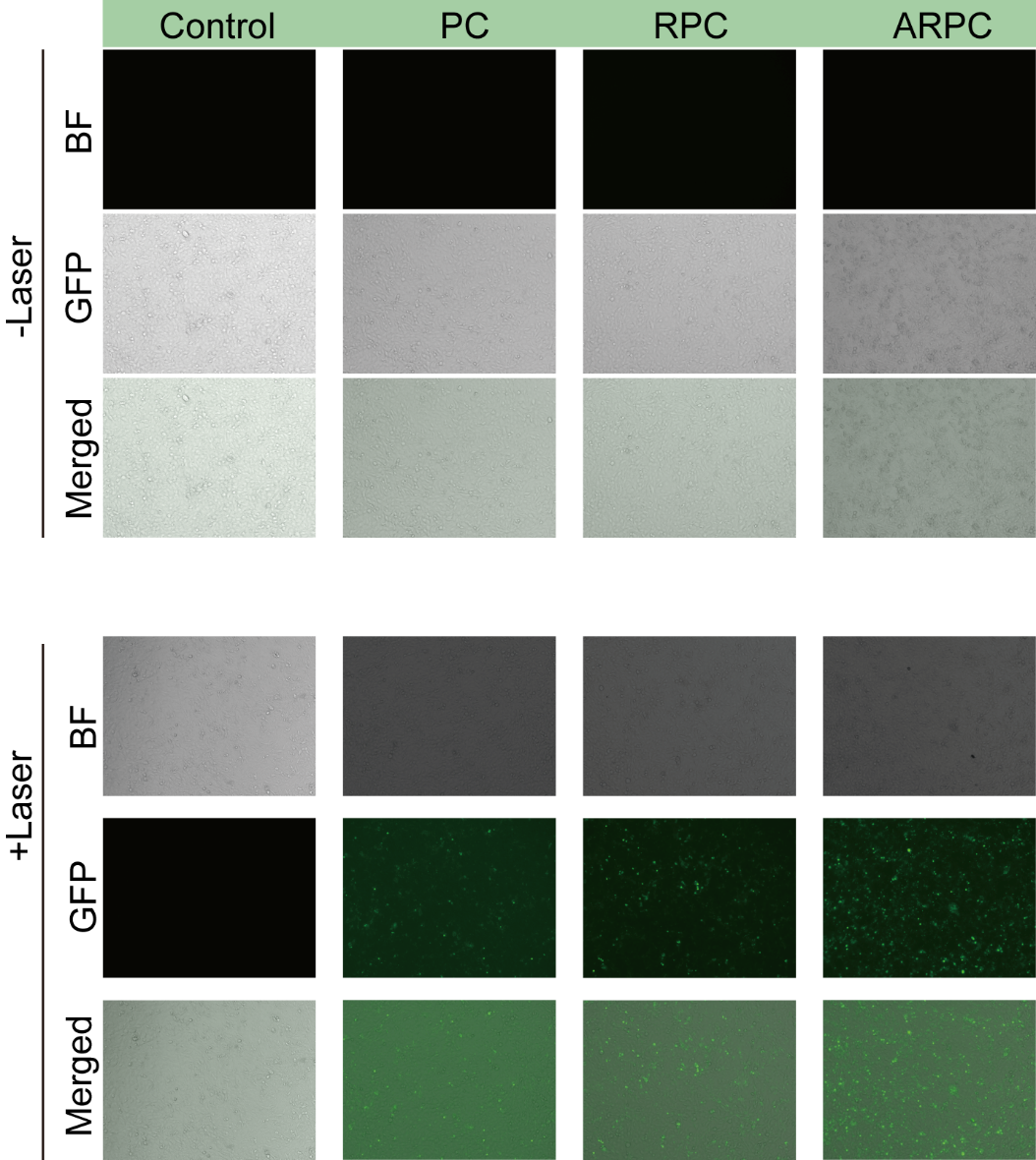


**Figure S32.** Each individual fluorescence channel of GFP.


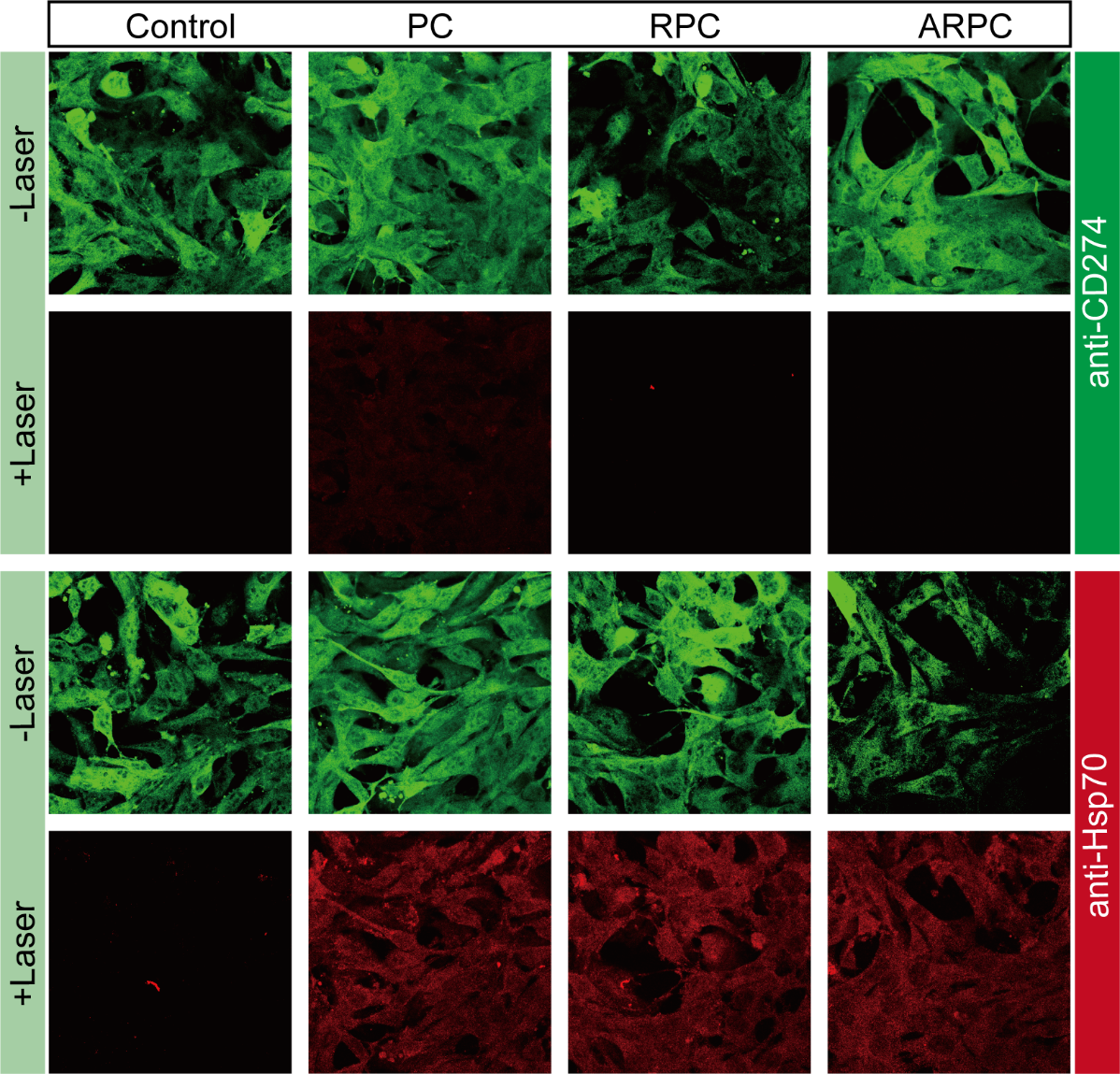


**Figure S33.** Each individual fluorescence channel of anti-CD274 (green) and anti-Hsp70 (red) in SCC7 cells.


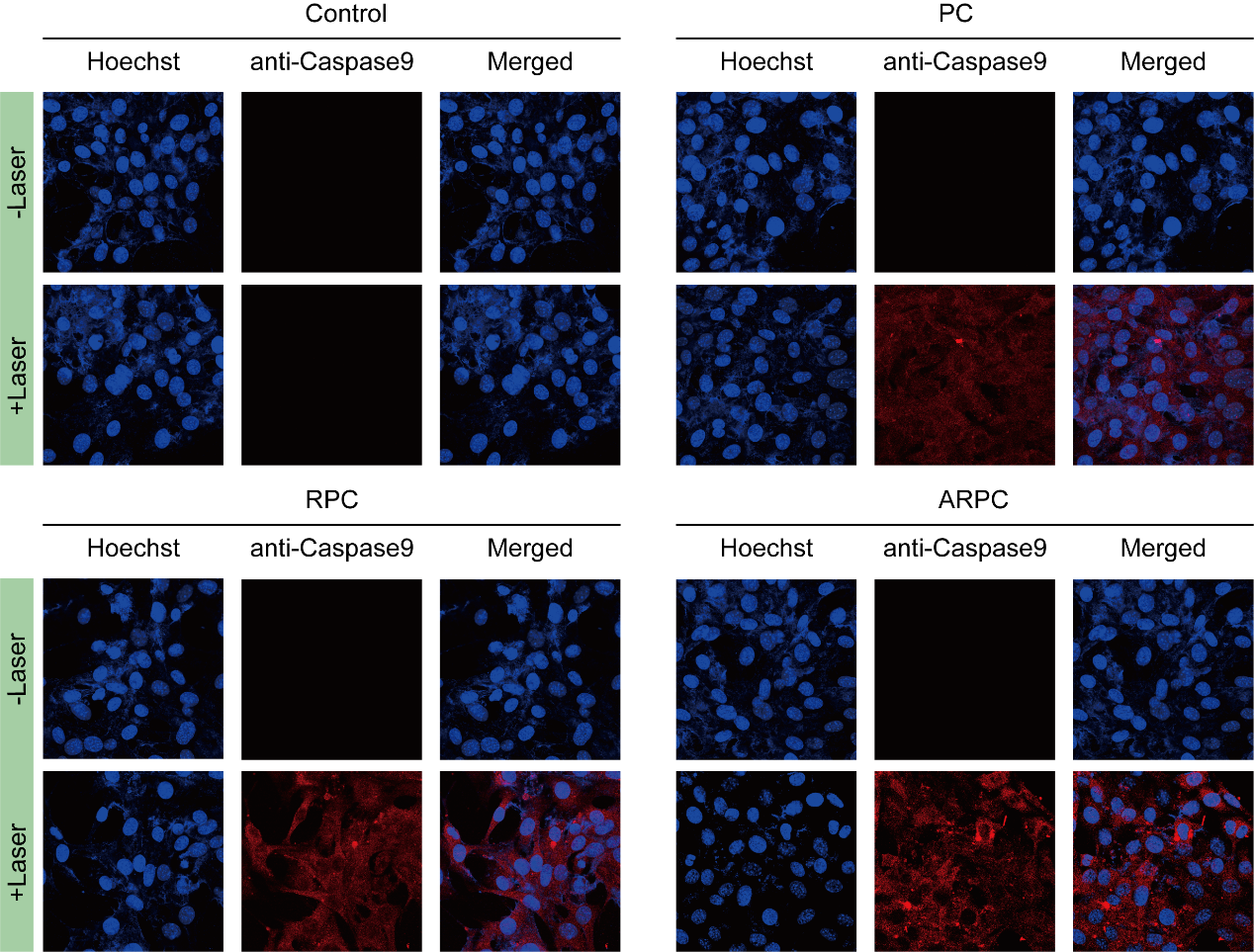


**Figure S34.** Each individual fluorescence channel of anti-Cas9 (red) in SCC7 cells.

**Table S3.** Adapter information for DNA-Seq.

| Adapter | Adapter Sequence |
| --- | --- |
| 5’ Adapter | 5’-AATGATACGGCGACCACCGAGATCTACACTCTTTCCC  TACACGACGCTCTTCCGATCT -3’ |
| 3’ Adapter | 5’-GATCGGAAGAGCACACGTCTGAACTCCAGTCAC(6-base index)ATCTCGTATGCCGTCTT-3’ |

**Table S4.** Detailed statistical quality control data (Sample ID: Sample name; Raw Data (Gb): Total amount of raw sequencing data; Raw Reads: Total number of paired-end reads in the raw sequencing data; Raw Data Q20: Percentage of bases in the raw sequencing data with a Phred score greater than 20; Raw Data Q30: Percentage of bases in the raw sequencing data with a Phred score greater than 30; N Rate: Proportion of sequences containing 'N' in the raw data; Adapter: Proportion of sequences containing adapter sequences in the raw data; Error Rate: Average error rate of all bases in the raw sequencing data; Clean Data (Gb): Amount of valid sequences after quality control; Clean Reads: Total number of paired-end reads in the cleaned data; Clean Data Q20: Percentage of bases in the cleaned data with a Phred score greater than 20; Clean Data Q30: Percentage of bases in the cleaned data with a Phred score greater than 30; GC: Percentage of bases that are either G or C out of the total number of bases; Effective Rate: Percentage of valid sequences obtained after quality control relative to the original raw data).

**Table S5.** Filtered results of the raw data (Adapter-related: The number of reads filtered out due to adapters and their proportion of the total raw reads. Containing N: The number of reads filtered out due to having more than 10% 'N' content in a single-end sequencing read and their proportion of the total raw reads. Low quality: The number of reads filtered out because the proportion of low-quality bases (quality score < 5) exceeded 50% of the read length and their proportion of the total raw reads. Clean reads: The number of clean reads obtained and their proportion of the total raw reads).

| Sample ID | Clean reads | Containing N | Low quality | Adapter related |
| --- | --- | --- | --- | --- |
| Control | 307234551 | 89854 | 0 | 1078504 |
| ARPC | 748799820 | 9922 | 0 | 3474664 |

**Table S6.** The concise correspondence between base calling and Phred scores in Illumina Casava 1.8 version.

| Phred score | Incorrect base calling | Base calling accuracy rate | Q-score |
| --- | --- | --- | --- |
| 10 | 1/10 | 90% | Q10 |
| 20 | 1/100 | 99% | Q20 |
| 30 | 1/1000 | 99.9% | Q30 |
| 40 | 1/10000 | 99.99% | Q40 |


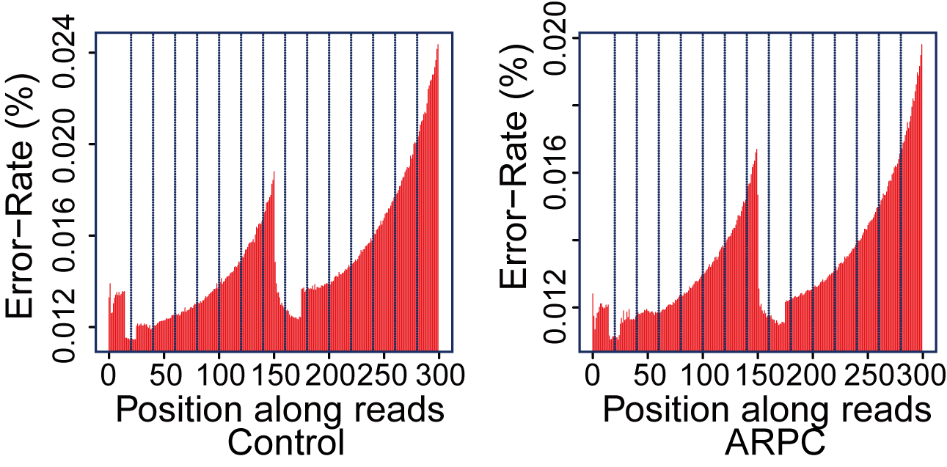


**Figure S35.** The base error rate distribution plot for Control and ARPC.


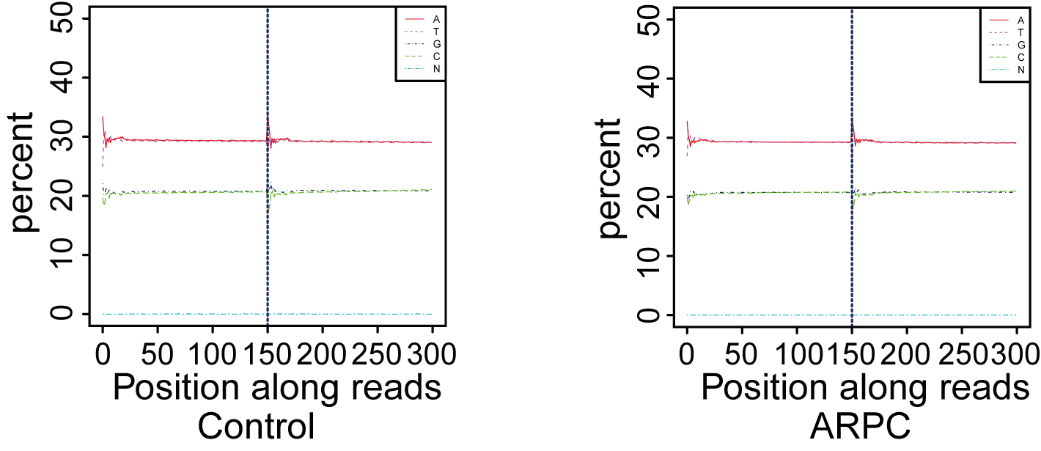


**Figure S36.** The GC content distribution plot for Control and ARPC.


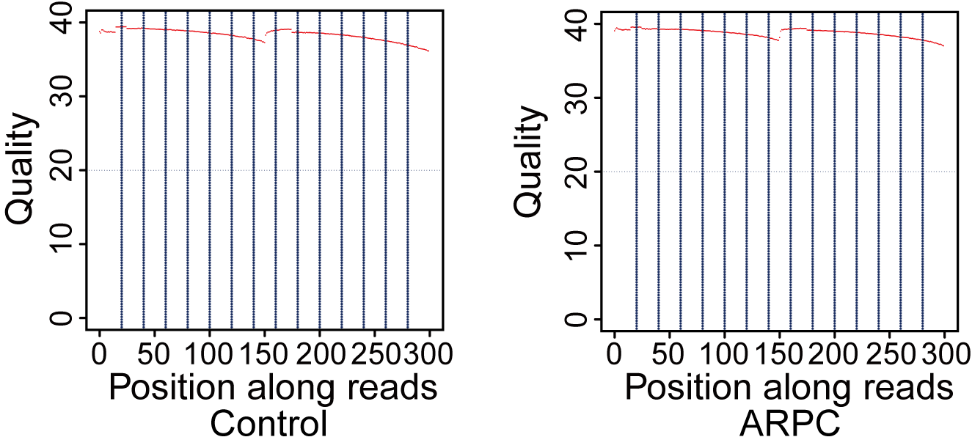


**Figure S37.** The base quality distribution plot for Control and ARPC.

**Table S7.** Sequencing Depth and Coverage Statistics (ref_name: Reference sequence ID; query_name: Sample ID; avg_depth: Average sequencing depth; coverage ≥ nX: Coverage with sequencing depth greater than a specified value (n); map_rate: Alignment rate of sample sequencing data to the reference sequence; mismatch_rate: Mismatch rate of sample sequencing data to the reference sequence; total_base: Total amount of sequencing data; total_map_base: Amount of mappable data; total_mismatch: Amount of mismatched data).

**Table S8.** The number of SNPs in exonic regions of the genome and different types of coding regions (Sample ID: Sample name; Non-synonymous_SNV: Non-synonymous mutation; Stopgain: A single-base substitution results in the codon being changed to a stop codon; Stoploss: A single-base substitution results in a stop codon being changed to a non-stop codon; Synonymous_SNV: Synonymous mutation, where the amino acid encoded by the variant site remains unchanged; unknowns: Unknown functional sites due to the inadequacy of the gene structure annotation database used for annotation).

| Sample ID | Nonsynonymous-SNV | Stopgain-SNV | Stoploss-SNV | Synonymous-SNV | unknown |
| --- | --- | --- | --- | --- | --- |
| Control | 17869 | 215 | 43 | 25915 | 531 |
| ARPC | 17179 | 207 | 42 | 25574 | 509 |

**Table S9.** The number of SNPs in functional genomic regions and different types of coding regions (Sample ID: Sample name; Downstream: 1Kb region downstream of the transcription termination site; Exonic: Exonic region; Exonic/splicing: Exonic region / 10bp splicing junction region; Intergenic: Intergenic region; Intronic: Intronic region; Splicing: 10bp splicing junction region; Upstream: 1Kb region upstream of the transcription start site; Upstream/downstream: 1Kb region upstream of the transcription start site/1Kb region downstream of the transcription termination site; UTR3: 3'UTR region; UTR5: 5'UTR region; UTR5/UTR3: 5'UTR region/3' UTR region).

**Table S10.** The distribution of InDel numbers in exonic regions of the genome and in different types of coding regions (Sample ID: Sample name; Frameshift deletion: A deletion that alters the reading frame of the encoded protein, with the deletion length not being a multiple of 3; Frameshift insertion: An insertion that alters the reading frame of the encoded protein, with the insertion length not being a multiple of 3; Non-frameshift deletion: A deletion that does not alter the reading frame of the encoded protein, with the deletion length being a multiple of 3; Non-frameshift insertion: An insertion that does not alter the reading frame of the encoded protein, with the insertion length being a multiple of 3; Stopgain: A single-base substitution that changes the codon to a stop codon; Stoploss: A single-base substitution that changes a stop codon to a non-stop codon; Unknown: Unknown functional sites due to the inadequacy of the gene structure annotation database used for annotation).

**Table S11.** The distribution of InDel numbers in different types of functional regions (Sample ID: Sample name; Downstream: 1 kb downstream of the transcription termination site; Exonic: Exon region; Exonic/Splicing: Exon region/splicing junction 10 bp region; Intergenic: Intergenic region; Intronic: Intron region; Splicing: Splicing junction 10 bp region; Upstream: 1 kb upstream of the transcription start site; Upstream/Downstream: 1 kb upstream of the transcription start site/1 kb downstream of the transcription termination site; UTR3: 3’ UTR region; UTR5: 5’ UTR region; UTR5/UTR3: 5’ UTR region/3’ UTR region).

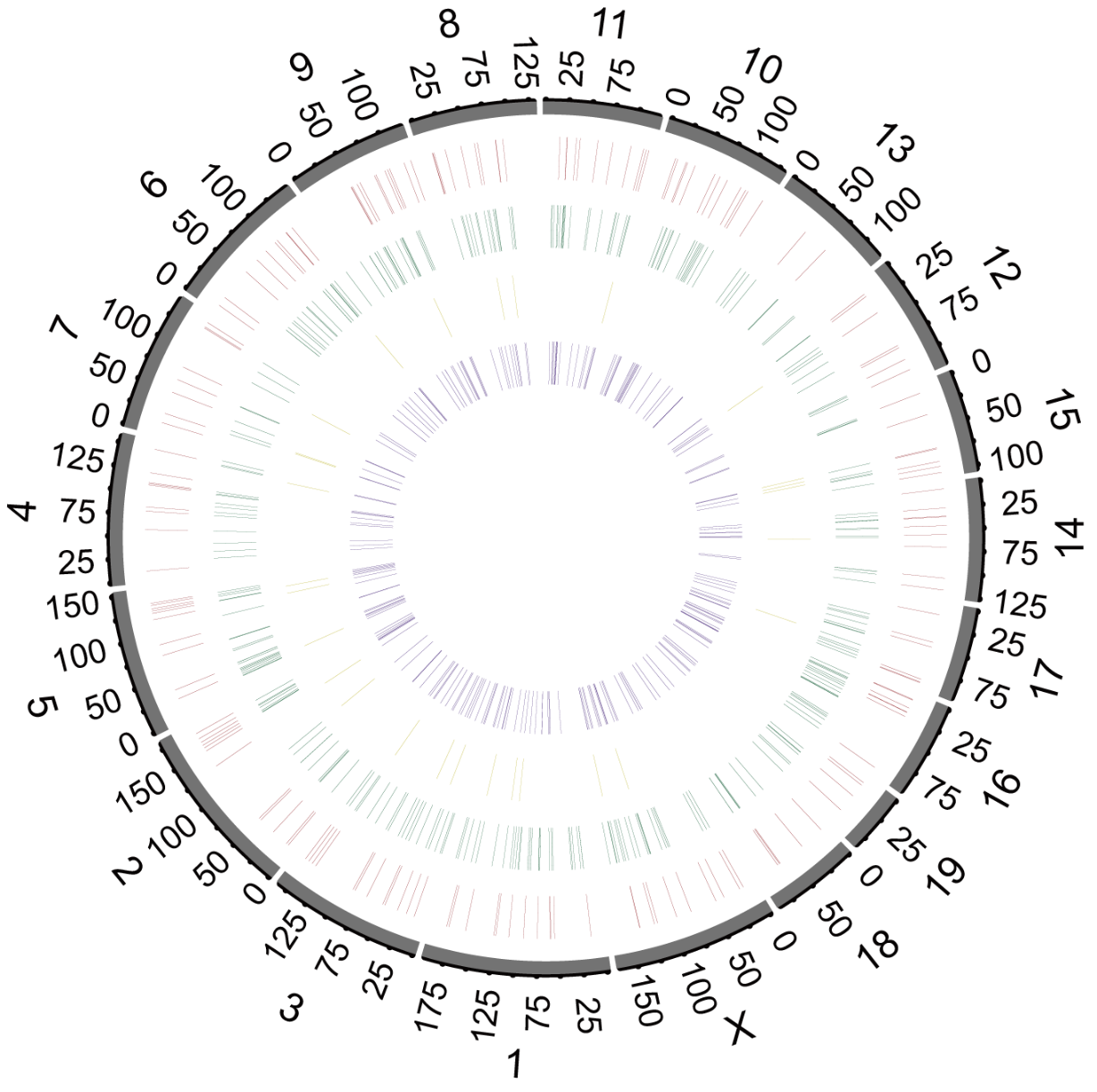


**Figure S38.** Detection of potential off-target sites in the PAM region of sgRNA (The Circos plot represents the AGG, TGG, CGG, and GGG sites from the outermost to the innermost circle).


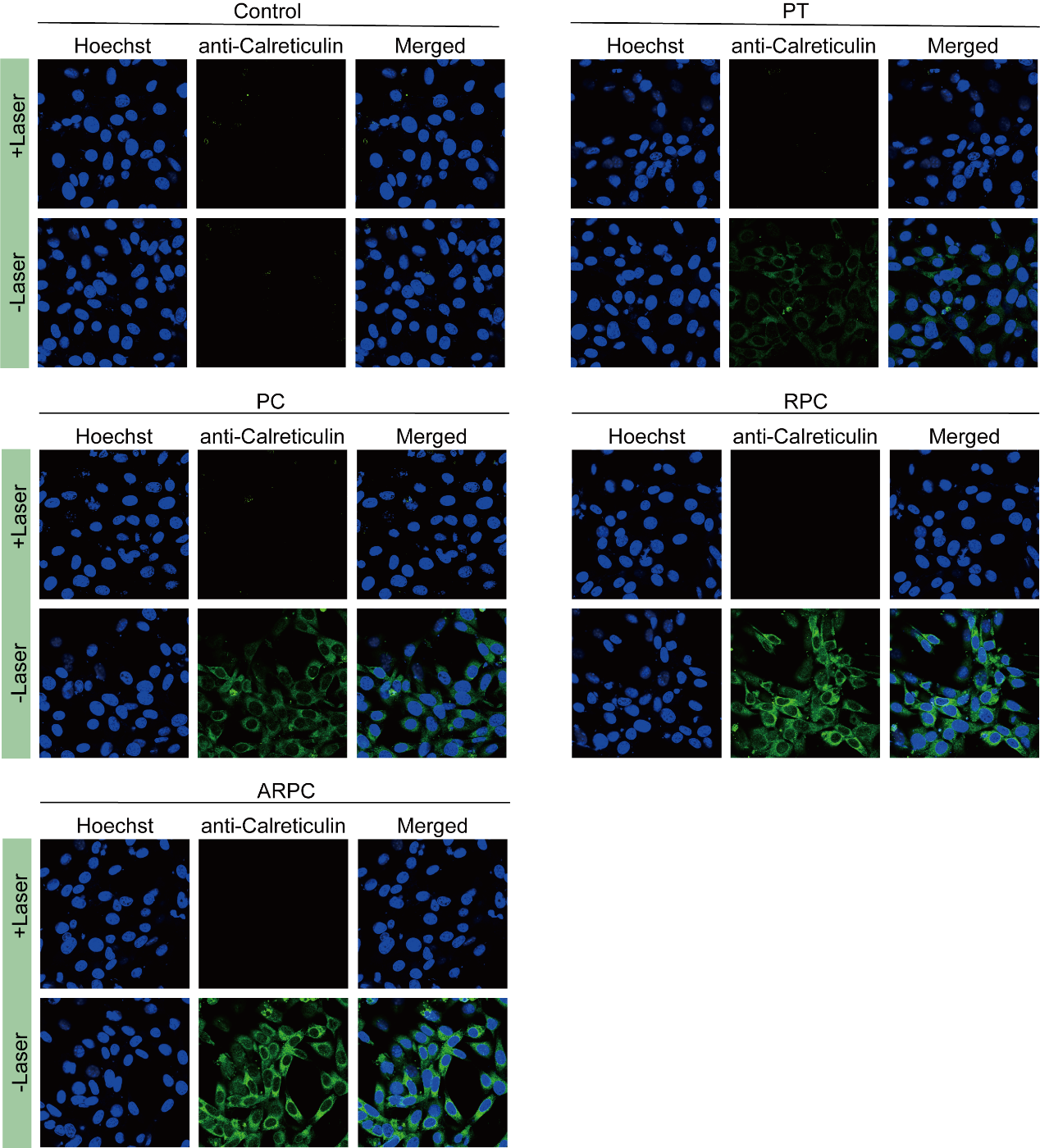


**Figure S39.** Each individual fluorescence channel of anti-Calreticulin (green) in SCC7 cells.


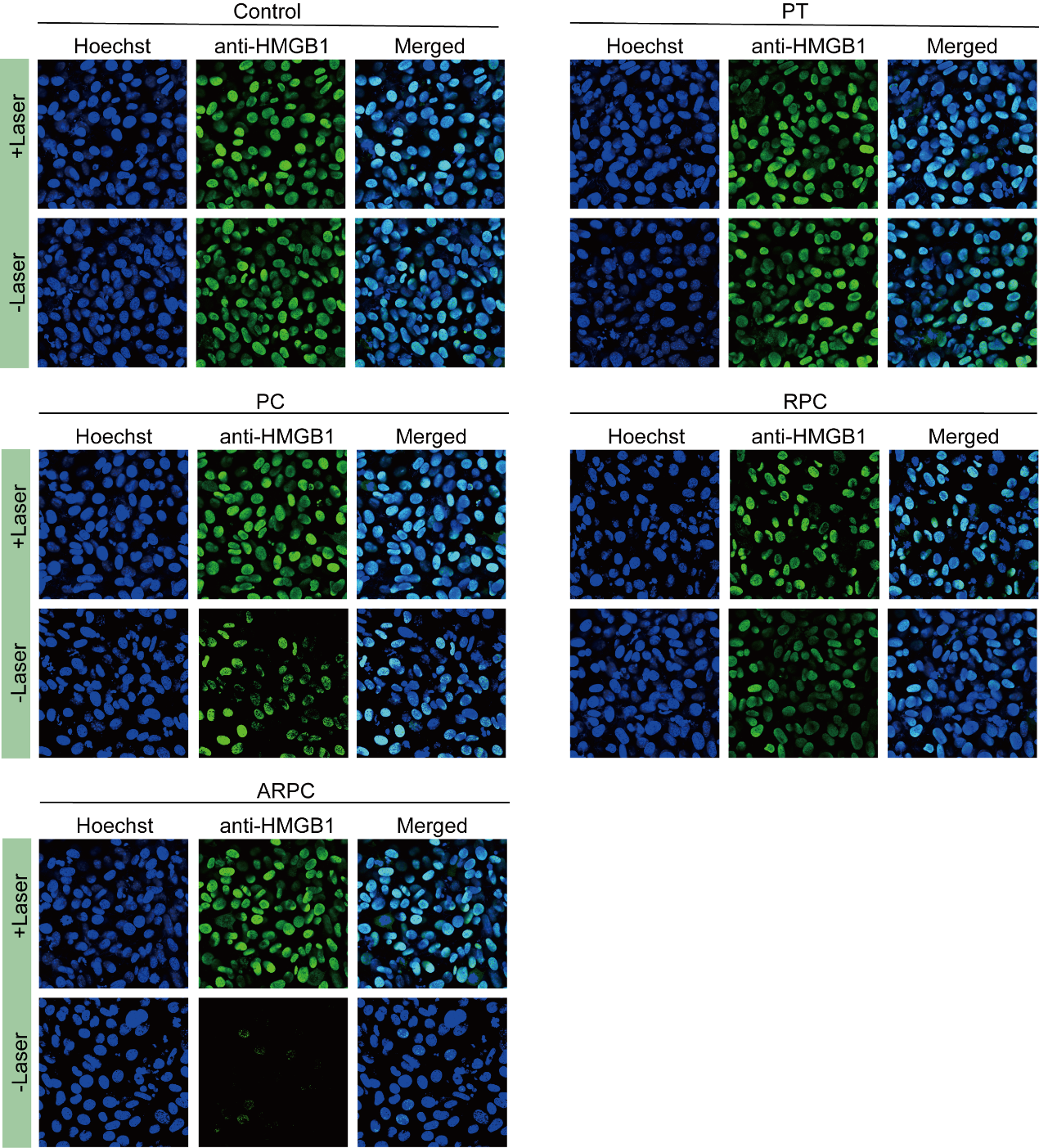


**Figure S40.** Each individual fluorescence channel of anti-HMGB1 (green) in SCC7 cells.


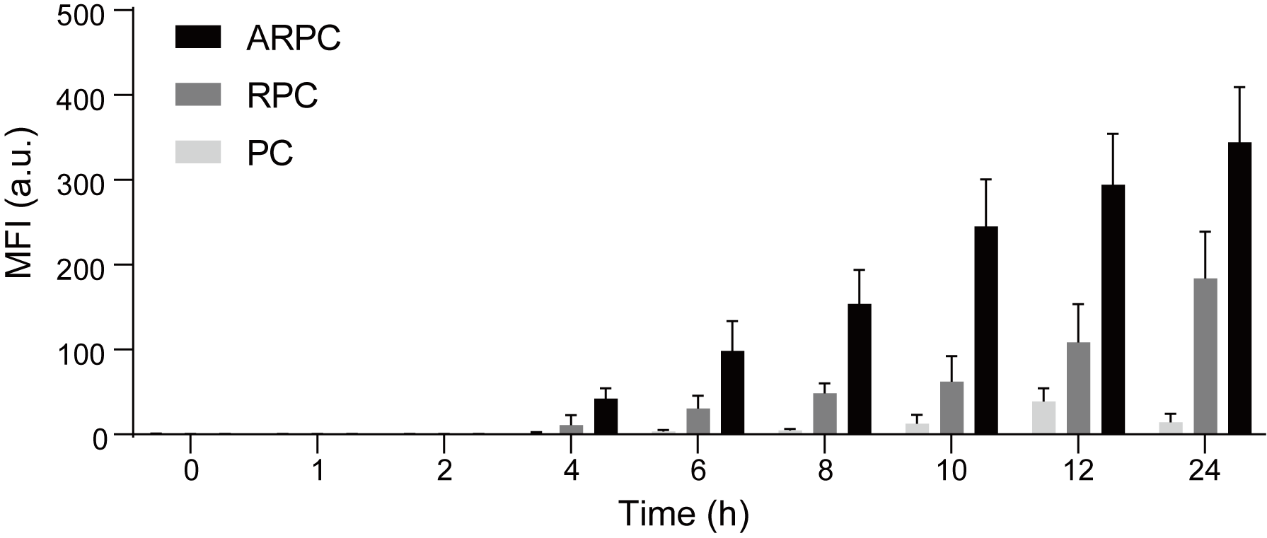


**Figure S41.** The inflorescence intensity of PC, RPC, ARPC measured by Image J. All data represents means ± SEM (n = 3).


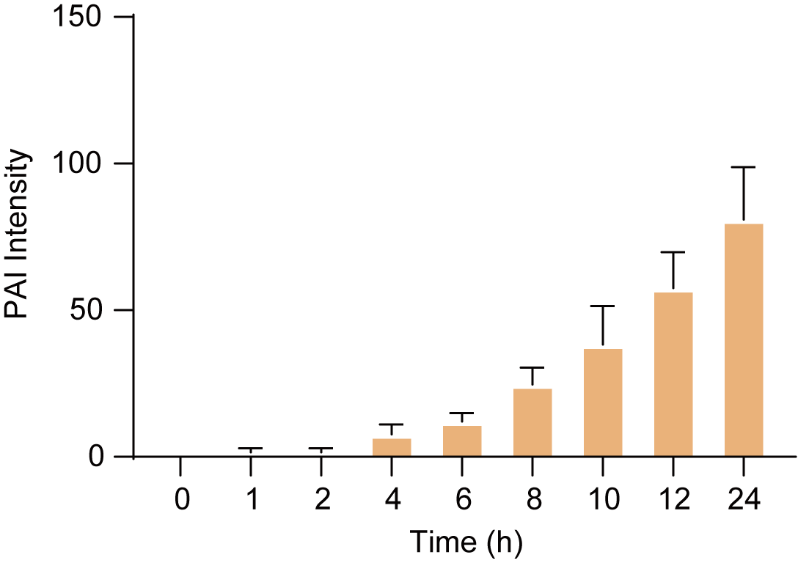


**Figure S42.** PAI intensity of ARPC measured by Image J. All data represents means ± SEM (n = 3).


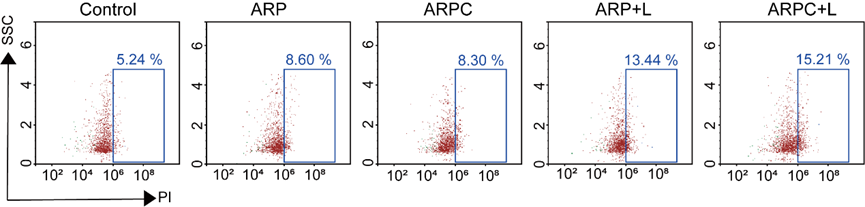


**Figure S43.** We stained the tumor tissues during the treatment process with PI and analyzed them by using a flow cytometer.


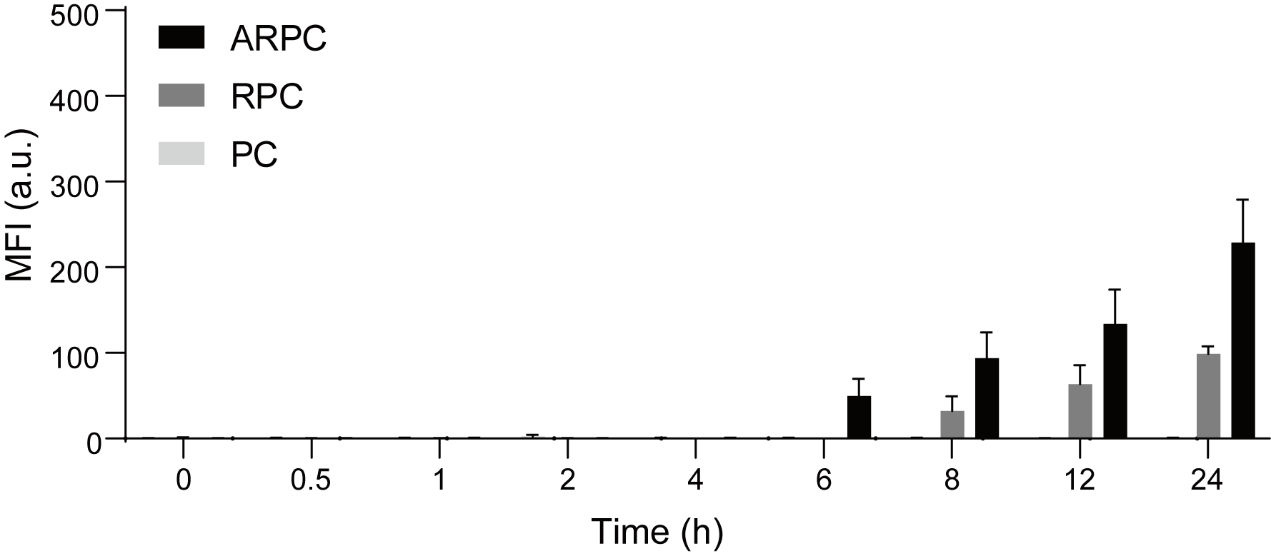


**Figure S44.** The inflorescence intensity of PC, RPC, ARPC measured by Image J. All data represents means ± SEM (n = 3).


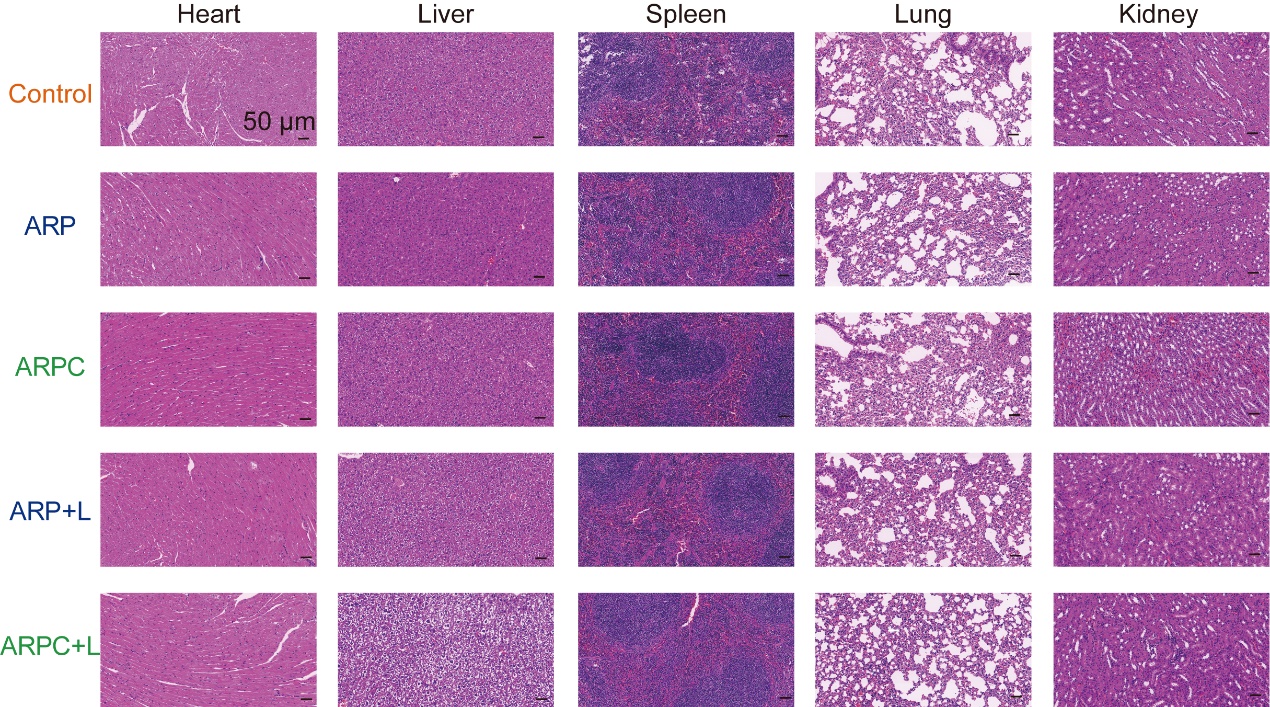


**Figure S45.** The images of H&E of major organ harvested by mice after Control, ARP, ARPC, ARP+L and ARPC+L, Scale bar = 50 μm. b) Hepatic and renal function test of ALT, AST, UREA, CREA.


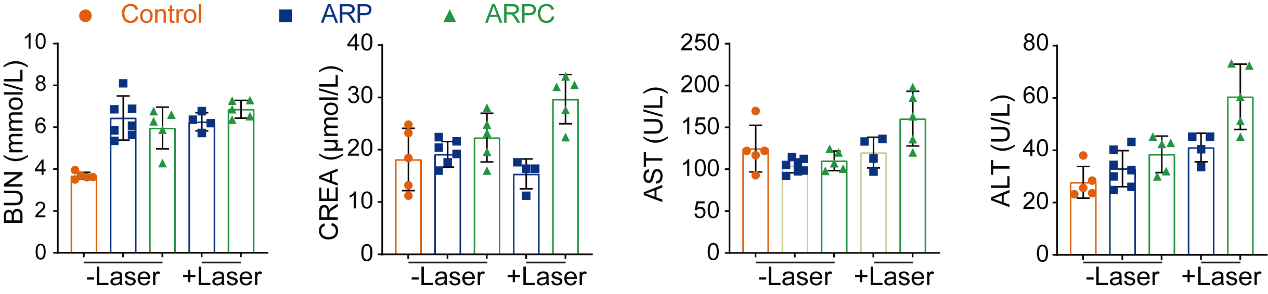


**Figure S46.** Hepatic and renal function test of ALT, AST, UREA, CREA.


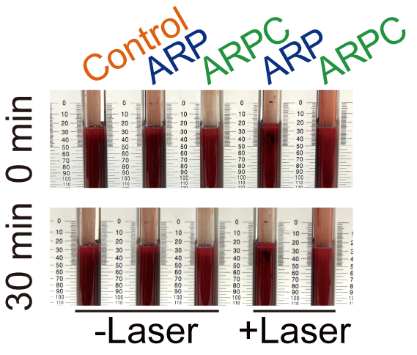


**Figure S47.** Erythrocyte sedimentation rate analysis post-treatment.


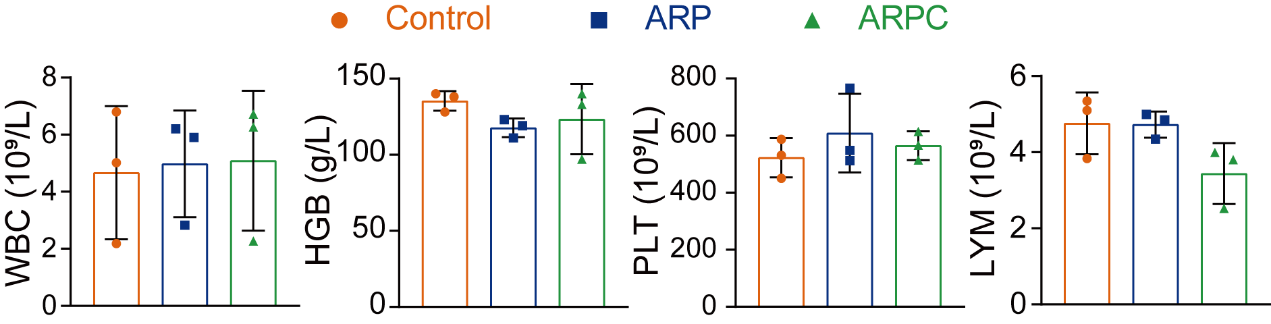


**Figure S48.** Complete blood count analysis post-treatment. All data represents means ± SEM (n = 3).
